# Association of Interprofessional Discharge Planning Using an Electronic Health Record Tool With Hospital Length of Stay Among Patients with Multimorbidity: A Nonrandomized Controlled Trial

**DOI:** 10.1001/jamanetworkopen.2022.33667

**Published:** 2022-09-28

**Authors:** Alexander Kutz, Daniel Koch, Sebastian Haubitz, Antoinette Conca, Ciril Baechli, Katharina Regez, Claudia Gregoriano, Fahim Ebrahimi, Stefano Bassetti, Jens Eckstein, Juerg Beer, Michael Egloff, Andrea Kaeppeli, Tobias Ehmann, Claus Hoess, Heinz Schaad, James Frank Wharam, Antoine Lieberherr, Ulrich Wagner, Sabina de Geest, Philipp Schuetz, Beat Mueller

**Affiliations:** 1 Medical University Department, Division of General Internal and Emergency Medicine, Cantonal Hospital Aarau, Aarau, Switzerland; 2 Division of Pharmacoepidemiology and Pharmacoeconomics, Department of Medicine, Brigham and Women’s Hospital and Harvard Medical School, Boston, Massachusetts; 3 Division of Gastroenterology, University Center for Gastrointestinal and Liver Diseases, St Clara Hospital and University Hospital, Basel, Switzerland; 4 Division of Internal Medicine, University Hospital Basel, Basel, Switzerland; 5 Faculty of Medicine, University of Basel, Basel, Switzerland; 6 Department of Medicine, Cantonal Hospital Baden, Baden, Switzerland; 7 Department of Medicine, Hospital Muri, Muri, Switzerland; 8 Department of Medicine, Hospital Zofingen, Zofingen, Switzerland; 9 Department of Medicine, Cantonal Hospital Muensterlingen, Muensterlingen, Switzerland; 10 Department of Medicine, Hospital Interlaken, Hospitals Frutigen Meiringen Interlaken, Interlaken, Switzerland; 11 Department of Medicine, Duke University and Duke-Margolis Center for Health Policy, Durham, North Carolina; 12 Federal Statistical Office, Neuchâtel, Switzerland; 13 National Institute for Cancer Epidemiology and Registration, National Agency for Cancer Registration, University of Zurich, Switzerland; 14 Nursing Science, Department of Public Health of Basel, Switzerland

## Abstract

**Importance:**

Whether interprofessional collaboration is effective and safe in decreasing hospital length of stay remains controversial.

**Objective:**

To evaluate the outcomes and safety associated with an electronic interprofessional-led discharge planning tool vs standard discharge planning to safely reduce length of stay among medical inpatients with multimorbidity.

**Design, Setting, and Participants:**

This multicenter prospective nonrandomized controlled trial used interrupted time series analysis to examine medical acute hospitalizations at 82 hospitals in Switzerland. It was conducted from February 2017 through January 2019. Data analysis was conducted from March 2021 to July 2022.

**Intervention:**

After a 12-month preintervention phase (February 2017 through January 2018), an electronic interprofessional-led discharge planning tool was implemented in February 2018 in 7 intervention hospitals in addition to standard discharge planning.

**Main Outcomes and Measures:**

Mixed-effects segmented regression analyses were used to compare monthly changes in trends of length of stay, hospital readmission, in-hospital mortality, and facility discharge after the implementation of the tool with changes in trends among control hospitals.

**Results:**

There were 54 695 hospitalizations at intervention hospitals, with 27 219 in the preintervention period (median [IQR] age, 72 [59-82] years; 14 400 [52.9%] men) and 27 476 in the intervention phase (median [IQR] age, 72 [59-82] years; 14 448 [52.6%] men) and 438 791 at control hospitals, with 216 261 in the preintervention period (median [IQR] age, 74 [60-83] years; 109 770 [50.8%] men) and 222 530 in the intervention phase (median [IQR] age, 74 [60-83] years; 113 053 [50.8%] men). The mean (SD) length of stay in the preintervention phase was 7.6 (7.1) days for intervention hospitals and 7.5 (7.4) days for control hospitals. During the preintervention phase, population-averaged length of stay decreased by −0.344 hr/mo (95% CI, −0.599 to −0.090 hr/mo) in control hospitals; however, no change in trend was observed among intervention hospitals (−0.034 hr/mo; 95% CI, −0.646 to 0.714 hr/mo; difference in slopes, *P* = .09). Over the intervention phase (February 2018 through January 2019), length of stay remained unchanged in control hospitals (slope, −0.011 hr/mo; 95% CI, −0.281 to 0.260 hr/mo; change in slope, *P* = .03), but decreased steadily among intervention hospitals by −0.879 hr/mo (95% CI, −1.607 to −0.150 hr/mo; change in slope, *P* = .04, difference in slopes, *P* = .03). Safety analyses showed no change in trends of hospital readmission, in-hospital mortality, or facility discharge over the whole study time.

**Conclusions and Relevance:**

In this nonrandomized controlled trial, the implementation of an electronic interprofessional-led discharge planning tool was associated with a decline in length of stay without an increase in hospital readmission, in-hospital mortality, or facility discharge.

**Trial Registration:**

isrctn.org Identifier: ISRCTN83274049

## Key Points

**Question:**

Is interprofessional discharge planning associated with safely reducing length of stay among medical inpatients?

**Findings:**

In this nonrandomized controlled trial including 493 486 hospitalizations at 7 intervention hospitals and 75 control hospitals, the implementation of an electronic interprofessional-led discharge planning tool was associated with a monthly reduction in length of stay by 0.9 hours, resulting in a cumulative benefit of 10.5 hours after the 1-year intervention phase. The intervention did not increase the risk of hospital readmission, in-hospital mortality, or facility discharge.

**Meaning:**

These findings support the broader implementation of discharge planning programs to reduce length of stay.

## Teaser

This nonrandomized controlled trial evaluates the outcomes and safety associated with an electronic interprofessional-led discharge planning tool designed to reduce length of stay among medical inpatients with multimorbidity.

## Introduction

Hospital length of stay (LOS) is a quality and reimbursement measure for efficient hospital management used by many health care systems. Worldwide, hospitals have aimed at reducing LOS to better match demand with capacity for new admissions and to improve turnaround of patients.^1^ Furthermore, prolonged LOS might be associated with increased inpatient complications and negative patient and staff experience.^2^ To overcome these problems, hospitals have specifically targeted patients, mostly those with multimorbidity, who tend to have longer LOS by reducing the number of long-stay beds, shifting care to the outpatient setting, and implementing policies such as discharge planning.^3^ In 2012, following a global trend, Switzerland classified patient hospital stays into diagnosis-related groups (SwissDRGs) with fixed reimbursements that further incentivized hospitals to reduce LOS to maintain operating margins. As in other countries, this raised safety concerns.^4^

Several approaches have been developed to optimize LOS, some focusing on aspects of patient management, such as clinical care,^5,6^ and others targeting staffing models^7,8^ and logistics of care coordination (eg, discharge planning and application of interprofessional care).^3,9,10,11^ Nevertheless, hospital discharge planning procedures have not been widely standardized, carrying the risk of poor communication, delayed assessments for postacute care demands, medication errors, or unprepared patient discharges.^12,13,14^

Health policy makers have called for the use of interprofessional collaboration as an essential element in improving safety of patient discharge and optimizing LOS,^15,16^ involving different health and social care professionals. Working together aims to overcome the fragmented, siloed, and burdensome provision of care that patients with medical complexity typically face.^17,18^ However, in Switzerland, interprofessional rounds are not necessarily standard, and evidence is still limited, especially regarding the effectiveness of interventions in older and multimorbid populations at risk for prolonged LOS and poor outcomes.^19^

To address this need, we developed a bundled hospital discharge planning tool that was embedded in the electronic medical records (EMRs) for use by the existing interprofessional ward teams. We hypothesized that this multifaceted intervention to shorten waiting times during hospital stay through an improved interprofessional collaboration would be associated with decreased LOS without increased hospital readmission and other adverse patient outcomes among medical inpatients with multimorbidity.

## Methods

### Overview

This nonrandomized controlled trial was part of the National Research Program Smarter Health Care, launched by the Swiss National Science Foundation to promote innovative health services research and to tackle practical challenges of caring for patients with multimorbidity in Switzerland.^20^ As a quality improvement study, the institutional review board of Northwestern Switzerland approved the study and waived the need for individual informed consent by formulating a declaration of no objection. Eligible patients were informed by clinical staff and an information flyer about their study participation within 24 hours after hospital admission. This study follows the Transparent Reporting of Evaluations with Nonrandomized Designs (TREND) reporting guideline.^21^

### Study Design and Participants

The Integrative Hospital Treatment in Older Patients to Benchmark and Improve Outcome and Length of Stay (In-HospiTOOL) study was an investigator-initiated, multicenter quality improvement study. The trial protocol appears in Supplement 1. It investigated whether the implementation of an electronic interprofessional-led discharge planning tool (In-HospiTOOL) would be associated with decreased LOS compared with standard discharge planning without negatively affecting relevant safety outcomes. Using a controlled interrupted time series design, this study compared outcomes among patients in hospitals that implemented In-HospiTOOL (intervention group) vs hospitals without such an additional intervention (control group). The overall 24-month study period was divided into a 12-month preintervention phase, from February 2017 to January 2018, and a 12-month intervention phase, from February 2018 to January 2019. From August 2017 to January 2019, patients were followed up for 30 days after hospital admission by structured telephone interviews to assess patient-reported outcomes, such as activities of daily living and satisfaction with care, among others not discussed in this article (eFigure 1 in Supplement 2). The rationale for this study, design details, and eligibility features have been previously published.^22^

Of 10 invited hospitals, 7 agreed to participate in the study. Declining hospitals were not asked to provide reasons for not participating but were not different in terms of patient characteristics and setting of care when compared with participating hospitals. Invitations were extended given prior scientific collaboration and the availability of the same EMR to facilitate technical implementation of the tool. Although not using the same EMR, participation of the University Hospital Basel was included because it would increase the generalizability of the findings. Control hospitals followed their individual standard discharge planning strategies.

The 7 intervention hospitals were secondary and tertiary care hospitals and included the University Clinic in Aarau, the University Hospital in Basel, the cantonal hospitals in Baden and Muensterlingen, and the hospitals in Interlaken, Muri, and Zofingen. The remaining 75 secondary and tertiary care hospitals in Switzerland with comparable characteristics were included in the control group.

Consecutively admitted medical patients aged 18 years or older with at least 2 diagnoses in need of acute treatment were prospectively included in the study. This requirement was chosen to better address the level of multimorbidity in the target study population. Eligible patients were admitted via the emergency department and were required to be hospitalized for at least 24 hours. Patients treated in an intensive care unit (ICU) only were excluded, given that the intervention was not applied to ICU patients. Patients with outlier hospitalization stays (ie, >100 days) were excluded from the analysis. The same eligibility criteria were retrospectively applied to patients among control hospitals.

### The In-HospiTOOL

**Figure 1.**
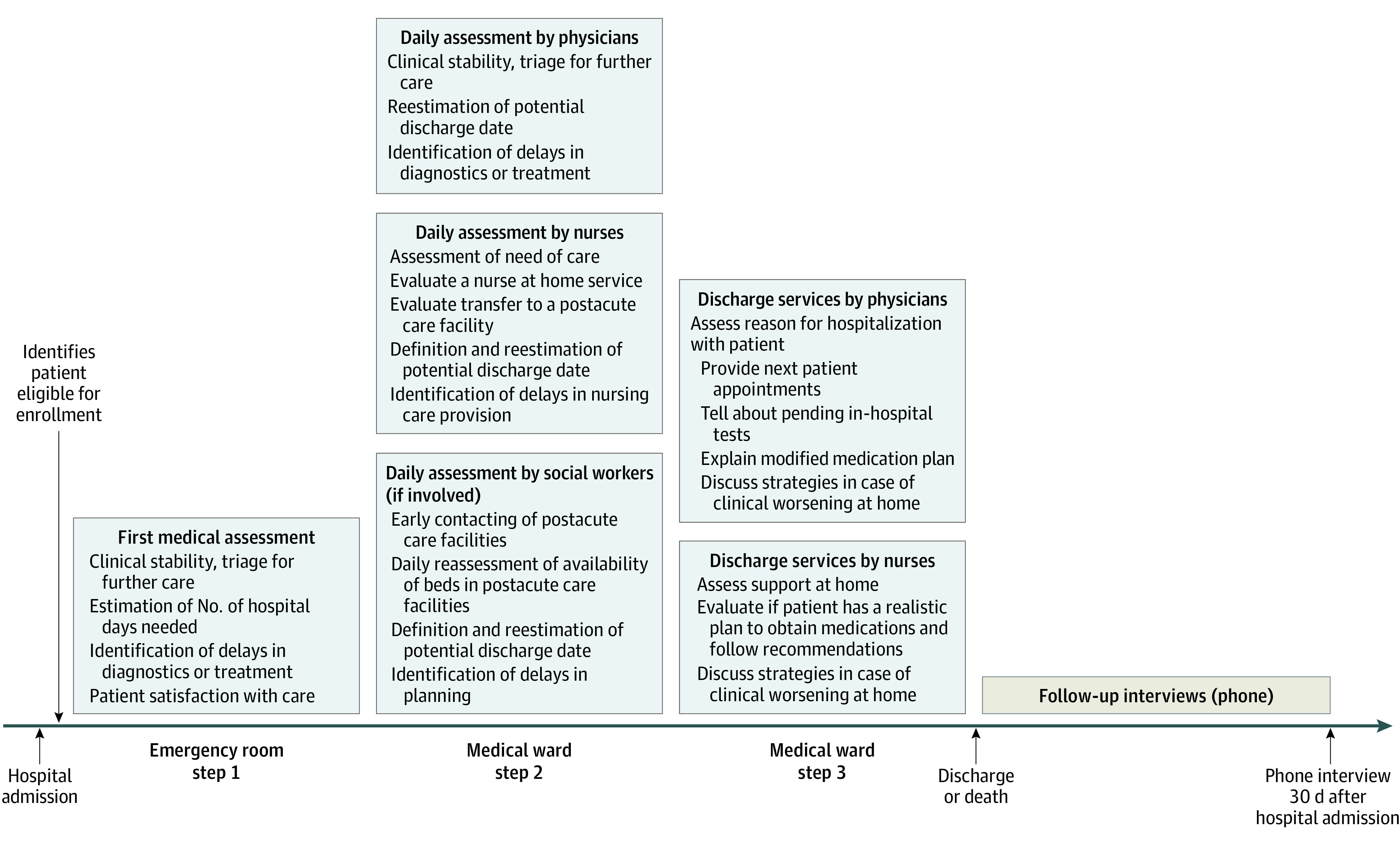
Activities of the Integrative Hospital Treatment in Older Patients to Benchmark and Improve Outcome and Length of Stay Study Along the Patient Transition Pathway Step 1 included an early discharge date estimation done by the emergency care team; step 2 involved a systematic assessment of delaying factors (diagnostics and therapy) during hospital stay, a modification of the potential discharge date, and an early assessment and contacting of postacute care facilities; step 3, physicians and nurses performed a timely discussion of pending in-hospital tests, out-of-hospital appointments, changes in medication plan, and strategies in case of clinical worsening at home with patients and relatives. Patient comprehension was assessed using the teach-back methodology. Information was recorded by either physicians, nurses, or social workers and was immediately available to all involved professionals using the EMR.

The In-HospiTOOL is an EMR-based application that standardizes interprofessional communication and was designed for team-based rounding at patient’s bedside. The tool was developed based on a sounding board consensus involving national stakeholders from the health care, information technology, and governmental sectors. The tool encompassed a bundle of interventions performed by physicians, nurses, and social workers that took place in the emergency department (step 1), at the medical ward (step 2), and before hospital discharge (step 3). In step 1, the main factors for improving discharge planning were an early estimation of the potential discharge date and the identification of potential delaying factors in diagnostics or treatment. The information from the emergency department was immediately applicable to all involved professions (eg, at the ward) on an EMR application. In step 2, physicians reevaluated the patient’s potential discharge date daily based on clinical condition, diagnostics, and therapeutics; nurses assessed any postacute care needs using the postacute care discharge (PACD) score^23^ during the first day on medical ward; and, if needed, social workers were required to contact postacute care facilities early to reduce transfer waiting times. In step 3, physicians and nurses instructed patients about the most important findings during the hospitalization, pending in-hospital diagnostics, changes to their medication plan, strategies in case of clinical worsening at home, and next clinical appointments following elements of the BOOST (Better Outcomes by Optimizing Safe Transitions) checklist.^13^ Details of the tool are illustrated and described in Figure 1.

### Interventions

We evaluated the implementation process of the intervention using the Reach, Effectiveness, Adoption, Implementation, Maintenance (RE-AIM) framework. Table 1 provides domain definitions.^24,25^ This process evaluation allows insight into how study interventions were implemented and might be optimized to aid future dissemination.

**Table 1.** Definition of RE-AIM Domains Along the Study Intervention

RE-AIM domain	Definition in In-HospiTOOL implementation evaluation
Reach	Proportion of hospitals that agreed to participate as an intervention hospital during the study 7 of 10 invited hospitals agreed to participate Designation of ≥1 physician, 1 nurse, and 1 social worker as local team leaders in each hospital Team leaders were instructed in procedural details of the study, received training on how to perform structured interprofessional team-based bedside rounding using the tool, and then disseminated this information among their colleagues Training of team leaders was accomplished through onsite visits or phone conferences at least every month To guarantee a standardized education of the local staff, we provided a teaching video to all study sites, where the appropriate usage of the tool meticulously described (BZP,^24^ 2018)
Effectiveness	Primary and secondary (balancing) outcomes Primary (hospital length of stay) Secondary (30-day all-cause hospital readmission, all-cause in-hospital mortality, facility discharge)
Adoption	Extent to which caregivers actually adopted the intervention in the study (showed compliance with the intervention) The implementation and progress of using the tool (frequency of tool use) were closely monitored by local team leaders
Implementation	Extent to which the intervention is implemented as intended, including implementation barriers and facilitators All team leaders continuously coached medical ward staff during the intervention period and provided real-time feedback to them to standardize the process and diminish individual variability during ward rounds In addition to the assessment of frequency in tool use, a close monitoring by team leaders allowed intervention to improve alignment with the key principles and study goals, if appropriate The local social work leader rounded with the care team to detect postacute care demands early
Maintenance	Extent to which the intervention is intended to be sustained over time and become institutionalized Meetings with stakeholder and providing support in programmatic incorporation of the tool along EMR

Abbreviations: In-HospiTOOL, Integrative Hospital Treatment in Older Patients to Benchmark and Improve Outcome and Length of Stay; EMR, electronic medical records; RE-AIM, Reach, Effectiveness, Adoption, Implementation, Maintenance.

Although not monitored by the study team, control hospitals followed their own discharge planning procedures, usually (but not mandatorily) including an interdisciplinary assessment of patient’s need for follow-up care after leaving the hospital and arrangements for that care.

### Data Collection

To provide regular feedback regarding frequency of tool use, all intervention hospitals implemented a structured data export that was sent monthly to the involved hospital staff. These data were sourced from the hospital-specific EMR. To assess patient covariates and outcomes of interest, for both intervention and control hospitals, anonymous inpatient claims data were obtained from the Federal Statistical Office in Neuchâtel, Switzerland. Among patients who were transferred between acute care hospitals, hospital stays were combined into a single episode of care, and the patient outcome was attributed to the first hospital.

### Measures and Outcomes

As a measure of adherence, the frequency of tool use was defined as the proportion of patients for whom the tool was used in the numerator and eligible patients in the denominator. Tool use was defined as filling at least 1 element of the tool; however, step 2 was mandatory because it included the core elements of the intervention with a daily reassessment of discharge-relevant aspects by all involved professionals, as described previously.

The primary outcome was LOS, defined as days spent in the hospital during the index hospitalization. The index hospitalization included all readmissions into the index hospital within 18 days after discharge from the index hospitalization if the main reason for rehospitalization was related to the main diagnosis from the index hospitalization. According to the SwissDRG definition, every readmission into the same hospital after 18 days from discharge or any readmission into another hospital was defined as a new index hospitalization.^26^ Thus, a single patient may have more than 1 index admission during the study period. Hospitalizations that crossed from 1 study phase to another or that went past the study end date were attributed to the study phase applicable during hospital admission.

Secondary outcomes included any all-cause hospital readmission within 30 days after discharge, all-cause in-hospital mortality, and facility discharge (ie, long-term care facility, convalescent home, or rehabilitation clinic). Regarding hospital readmission, both planned and emergency readmissions were included in the analysis. Out-of-hospital mortality data were not available in the claims data set and could not be linked to the national death registry. We did not evaluate the relative outcomes associated with the separate elements of this bundled intervention. Blinding of patients, staff, and study personnel was not feasible due to the hospital ward-based nature of the intervention.

### Statistical Analysis

We descriptively examined patient and hospital characteristics of index hospitalizations for intervention and control hospitals for the preintervention phase and the season-matched intervention phase. The index hospitalization was the unit of analysis, and the statistical analysis was on an intention-to-treat basis.

To analyze population-averaged trends in LOS, 30-day hospital readmission, in-hospital mortality, and facility discharge, we performed segmented regression analyses of interrupted time-series data from February 2017 through January 2019. To model trends in LOS, we used a multivariable mixed-effects linear regression, including study time, modeled continuously as a linear spline with 1 knot location chosen at the start of the intervention phase (February 2018). In addition, we added a parameter to assess a change in level in LOS at the start of the intervention phase. The model included random effects for hospital and patient-specific intercept and slope to allow for individual trends for each hospital and patient, which improved models’ fit over random intercept only models, based on log likelihood ratio tests, with statistical significance set at *P* < .05. At the patient-level, the model was adjusted for age, sex, housing conditions, level of health care insurance coverage, Elixhauser comorbidity index, and the Hospital Frailty Risk Score.^27^ Time coefficients can be interpreted as monthly mean change in hours of hospitalization.

To explore whether the intervention was associated with patient safety, we assessed the previously mentioned secondary outcomes using multivariable mixed-effects logistic regression analyses following the same specification as described for the linear model. Resulting time coefficients can be interpreted as monthly mean change in percentage. We censored patients who died during hospitalization when assessing hospital readmission and facility discharge. There were no missing data for patient characteristics and study outcomes. Statistical significance was based on 95% CIs, and all *P* values are two-sided. All statistical analyses were performed using Stata version 17.0 (StataCorp).

## Results

### Index Hospital Admissions

Among 54 695 hospitalizations in 7 intervention hospitals, 27 219 (49.8%) were included during the preintervention phase and 27 476 (50.2%) during the intervention phase. Among the 75 nonintervention hospitals, 216 261 (49.3%) and 222 530 (50.7%) hospitalizations, respectively, served as controls (eFigure 2 in Supplement 2).

Baseline characteristics were generally similar between intervention and control hospitals (Table 2). In the 7 intervention hospitals, the median (IQR) age was 72 [59-82] years, and 14 400 patients (52.9%) were men during the preintervention phase; the median (IQR) age was 72 (59-82) years and 14 448 patients (52.6%) were men in the intervention phase. In the control hospitals, the median (IQR) age was 74 (60-83) years, and 109 770 patients (50.8%) were men in the preintervention phase; the median (IQR) age was 74 (60-83) years, and 113 053 patients (50.8%) were men in intervention phase. Of note, the proportion of patients treated in a university hospital was larger among intervention hospitals. Patients hospitalized in intervention hospitals were more likely to be male and being admitted from a nursing home. They were also hospitalized more frequently for a cardiovascular main diagnosis and had a slightly higher burden of comorbidities and frailty than patients in control hospitals. Between the preintervention and intervention phase, there were only minimal differences in baseline characteristics for both study groups.

**Table 2.** Baseline Characteristics of Patients

Characteristic	Patients, No. (%)			
	Intervention hospitals		Control hospitals	
	Preintervention phase, Feb 2017 to Jan 2018 (n = 27 219)	Intervention phase, Feb 2018 to Jan 2019 (n = 27 476)	Preintervention phase, Feb 2017 to Jan 2018 (n = 216 261)	Intervention phase, Feb 2018 to Jan 2019 (n = 222 530)
Sociodemographic				
Age, median (IQR), y	72 (59-82)	72 (59-82)	74 (60-83)	74 (60-83)
Gender				
Male	14 400 (52.9)	14 448 (52.6)	109 770 (50.8)	113 053 (50.8)
Female	12 819 (47.1)	13 028 (47.4)	106 491 (49.2)	109 477 (49.2)
Swiss residents	22 113 (81.2)	22 237 (80.9)	178 391 (82.5)	182 729 (82.1)
Private health insurance	5669 (20.8)	5678 (20.7)	48 625 (22.5)	48 177 (21.6)
Living at home before admission	22 467 (82.5)	22 632 (82.4)	192 116 (88.8)	197 881 (88.9)
Nursing home residents	1867 (6.9)	1905 (6.9)	9212 (4.3)	10159 (4.6)
Hospital				
Quarter of hospital admission				
Jan-Mar	7425 (27.3)	7333 (26.7)	56 915 (26.3)	59 450 (26.7)
Apr-Jun	6526 (24.0)	6711 (24.4)	51 398 (23.8)	53 256 (23.9)
Jul-Sep	6767 (24.9)	6702 (24.4)	52 613 (24.3)	53 784 (24.2)
Oct-Dec	6501 (23.9)	6730 (24.5)	55 335 (25.6)	56 040 (25.2)
Hospital teaching level				
University hospitals	6679 (24.5)	6220 (22.6)	31 153 (14.4)	32 800 (14.7)
Nonuniversity tertiary care hospitals	17 510 (64.3)	17 940 (65.3)	142 752 (66.0)	146 513 (65.8)
Secondary care hospitals	3030 (11.1)	3316 (12.1)	42 356 (19.6)	43 217 (19.4)
Level of morbidity				
Comorbidities				
Hypertension	14 613 (53.7)	14 704 (53.5)	110 018 (50.9)	115 012 (51.7)
Diabetes	5519 (20.3)	5692 (20.7)	42 330 (19.6)	44 072 (19.8)
Congestive heart failure	4063 (14.9)	4245 (15.4)	32 441 (15.0)	34 380 (15.4)
Coronary artery disease	6482 (23.8)	6554 (23.9)	47 393 (21.9)	49 576 (22.3)
Cerebrovascular disease	3368 (12.4)	3486 (12.7)	18 276 (8.5)	19 404 (8.7)
Chronic kidney disease	6314 (23.2)	6416 (23.4)	47 325 (21.9)	50 883 (22.9)
Liver disease	1161 (4.3)	1229 (4.5)	9319 (4.3)	9864 (4.4)
COPD	2661 (9.8)	2737 (10.0)	20 732 (9.6)	22 100 (9.9)
Cancer	3775 (13.9)	3739 (13.6)	28 434 (13.1)	29 142 (13.1)
Dementia	1634 (6.0)	1667 (6.1)	12 790 (5.9)	13 178 (5.9)
Groups of main diagnoses during hospital stay				
Cardiovascular	7445 (27.4)	7331 (26.7)	53 891 (24.9)	55 033 (24.7)
Respiratory	3610 (13.3)	3766 (13.7)	30 197 (14.0)	32 097 (14.4)
Oncology	2098 (7.7)	2155 (7.8)	15 718 (7.3)	15 858 (7.1)
Gastroenterology	2351 (8.6)	2324 (8.5)	17 275 (8.0)	17 732 (8.0)
Infectious disease	2018 (7.4)	2090 (7.6)	16 491 (7.6)	17 252 (7.7)
Elixhauser Comorbidity Index, mean (SD)^a^	3.0 (2.1)	3.1 (2.1)	2.8 (2.0)	2.9 (2.0)
Hospital Frailty Risk Score^b^				
<5, Low risk	17 698 (65.0)	17 636 (64.2)	143 041 (66.1)	145 014 (65.2)
5-15, Intermediate risk	8433 (31.0)	8729 (31.8)	66 582 (30.8)	70 072 (31.5)
>15, High risk	1088 (4.0)	1111 (4.0)	6638 (3.1)	7444 (3.3)

Abbreviation: COPD, chronic obstructive pulmonary disease.

^a^ Scores range from −7 to 12, with higher scores indicating greater comorbidity.

^b^ Scores range from 0 to 99, with higher scores indicating greater frailty.

### Frequency of Tool Use

During the intervention phase, the tool was used by either physicians, nurses, or both for mean (SD) 81.9% (38.5) of patients. The mean (SD) frequency of tool use among physicians was 77.6% (41.7), and among nurses, it was 75.5% (43.0%) (eFigure 3 in Supplement 2).

### Hospital Length of Stay

The mean (SD) LOS during the preintervention phase was 7.5 (7.4) days for control hospitals and 7.6 (7.1) days for intervention hospitals. It was 7.4 (7.2) days and 7.3 (7.0) days, respectively, during the intervention phase. The eTable in Supplement 2 shows crude estimates per study month.

**Figure 2.**
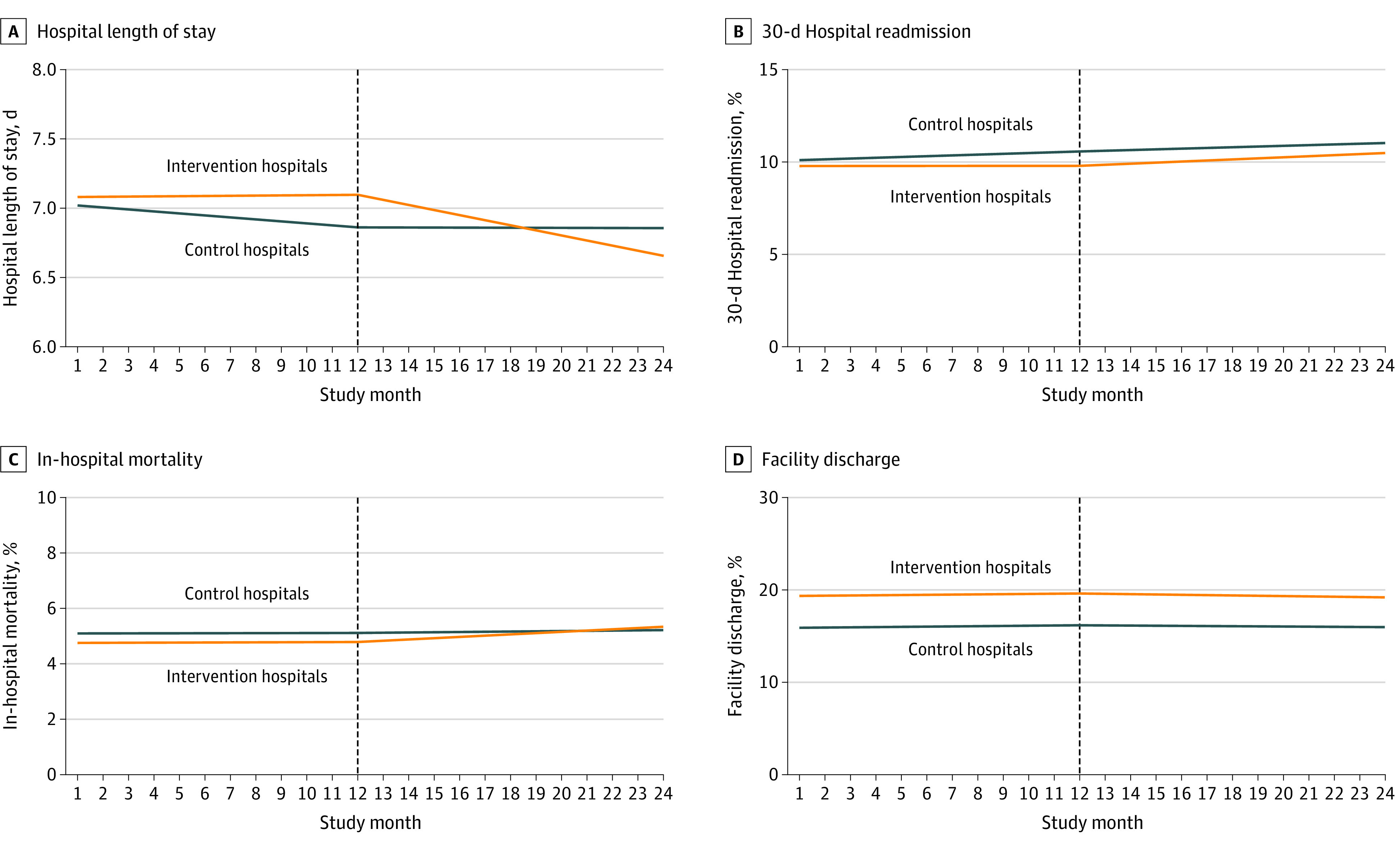
Trends in Hospital Length of Stay, 30-day Hospital Readmission, In-Hospital Mortality, and Facility Discharge for Intervention and Control Hospitals Before and After the Tool Implementation Graphs show population-averaged means for outcomes 1 year before and after the implementation of the intervention. The tool implementation in February 2018 is indicated with dashed vertical lines.

Over the year before implementing the tool, in control hospitals, LOS declined 0.344 hr/mo (95% CI, −0.599 to −0.090 hr/mo; *P* = .01), while there was no change in LOS among intervention hospitals (0.034 hr/mo; 95% CI, −0.646 to 0.714 hr/mo; *P* = .92) during the same time (Table 3 and Figure 2A). The difference in slopes between control and intervention hospitals was not statistically significant (*P* = .09). During the intervention phase, the decline ceased among control hospitals (−0.011 hr/mo; 95% CI, −0.281 to 0.260 hr/mo; *P* = .94; change from prior slope, *P* = .03), but it was more pronounced in intervention hospitals with a monthly reduction in LOS by 0.879 hours (95% CI, −1.607 to −0.150 hr/mo; change from prior slope, *P* = .04; difference in intervention phase slopes, *P* = .03). There was no evidence for a sudden change in level at the start of the intervention phase.

**Table 3.** Multivariable Random Slope Mixed-Effects Model of Changes in Hospital Length of Stay, 30-day Hospital Readmission, In-Hospital Mortality, and Facility Discharge for Intervention and Control Hospitals Between February 2017 and January 2019

Outcome and study phase^a^	Coefficient (95% CI)^b^	Slope (95% CI)^c^	*P* value		
			Slope differs from zero	Change in slope from prior slope	Difference in slopes (control vs intervention hospitals)
**Hospital length of stay^d^**					
Control hospitals, reference group					
Preintervention phase, Feb 2017 to Jan 2018	−0.344 (−0.599 to −0.090)	−0.344 (−0.599 to −0.090)	.01	.03	NA
Intervention phase, Feb 2018 to Jan 2019	0.334 (0.026 to 0.642)	−0.011 (−0.281 to 0.260)	.94		NA
Intervention hospitals					
Change in level in Feb 2017	1.086 (−26.399 to 28.571)	NA	NA	NA	NA
Intervention hospital by time interaction during preintervention phase, Feb 2017 to Jan 2018	0.378 (−0.332 to 1.089)	0.034 (−0.646 to 0.714)	.92	.04	.09
Intervention hospital by time interaction during intervention phase, Feb 2018 to Jan 2019	−1.247 (−2.160 to −0.334)	−0.879 (−1.607 to −0.150)	.02		.03
**30-d hospital readmission**					
Control hospitals, reference group					
Preintervention phase, Feb 2017 to Jan 2018	0.042 (0.011 to 0.074)	0.042 (0.011 to 0.074)	.01	.88	NA
Intervention phase, Feb 2018 to Jan 2019	−0.005 (−0.063 to 0.054)	0.038 (−0.023 to 0.099)	.22		NA
Intervention hospitals					
Change in level in Feb 2017	−0.277 (−2.264 to 1.710)	NA	NA	NA	NA
Intervention hospital by time interaction during pre-intervention phase, Feb 2017 to Jan 2018	−0.041 (−0.141 to 0.058)	0.001 (−0.094 to 0.096)	.99	.54	.15
Intervention hospital by time interaction during intervention phase, Feb 2018 to Jan 2019	0.061 (−0.129 to 0.251)	0.058 (−0.136 to 0.251)	.56		.85
**In-hospital mortality**					
Control hospitals, reference group					
Preintervention phase, Feb 2017 to Jan 2018	0.002 (−0.018 to 0.021)	0.002 (−0.018 to 0.021)	.88	.67	NA
Intervention phase, Feb 2018 to Jan 2019	0.007 (−0.025 to 0.039)	0.008 (−0.023 to 0.040)	.60		NA
Intervention hospitals					
Change in level in Feb 2017	−0.340 (−2.121 to 1.442)	NA	NA	NA	NA
Intervention hospital by time interaction during pre-intervention phase, Feb 2017 to Jan 2018	0.001 (−0.055 to 0.058)	0.003 (−0.051 to 0.057)	.92	.39	>.99
Intervention hospital by time interaction during intervention phase, Feb 2018 to Jan 2019	0.036 (−0.066 to 0.137)	0.045 (−0.052 to 0.143)	.36		.48
**Facility discharge**					
Control hospitals, reference group					
Preintervention phase, Feb 2017 to Jan 2018	0.024 (−0.023 to 0.072)	0.024 (−0.023 to 0.072)	.32	.24	NA
Intervention phase, Feb 2018 to Jan 2019	−0.041 (−0.110 to 0.028)	−0.017 (−0.088 to 0.055)	.65		NA
Intervention hospitals					
Change in level in February 2017	3.410 (−1.184 to 8.004)	NA	NA	NA	NA
Intervention hospital by time interaction during pre-intervention phase, Feb 2017 to Jan 2018	−0.001 (−0.148 to 0.146)	0.023 (−0.117 to 0.163)	.74	.58	.77
Intervention hospital by time interaction during intervention phase, Feb 2018 to Jan 2019	−0.017 (−0.233 to 0.200)	−0.034 (−0.257 to 0.188)	.76		.88

Abbreviation: NA, not applicable.

^a^ Time is treated continuously; coefficients and slopes are reported in monthly estimates (eg, change per month).

^b^ Model includes patient’s age, sex, housing condition, level of health care insurance coverage, Elixhauser comorbidity index, and Hospital Frailty Risk Score.

^c^ Slope during intervention phase can be derived by summing coefficient from preintervention phase and intervention phase.

^d^ Regression coefficients and slopes for hospital length of stay are provided in hours.

### Secondary Safety Outcomes

During the preintervention phase, the mean (SD) 30-day hospital readmission rate was 10.3% (12.5) among both control and intervention hospitals, and it was 10.4% (12.5) and 10.1% (12.3), respectively, during the intervention phase (eTable in Supplement 2). Mean in-hospital mortality was 5.2% (10.3) and 4.7% (10.4), respectively, during the preintervention phase, and 5.1% (10.1) and 4.9% (10.3), respectively, during the intervention phase. Discharge to a postacute care facility occurred in a mean (SD) 18.3% (23.1) and 20.3% (25.6), respectively, of hospitalizations during the preintervention phase and was comparable with the intervention phase (18.7% [23.2] and 20.3% [25.7], respectively). Segmented regression analyses revealed no differences in trends for all secondary outcomes of interest, except for a small increase in hospital readmission rate among control hospitals during the preintervention phase (slope, 0.042% per month; 95% CI, 0.011%-0.074%; *P* = .01) (Table 3 and Figure 2B-D).

## Discussion

In this multicenter study involving 493 486 hospitalizations, the implementation of an electronic interprofessional-led discharge planning tool embedded in the EMRs of 7 hospitals was associated with a decline in LOS among patients with multimorbidity compared with a control group of hospitals receiving standard discharge planning. There were no increases in hospital readmission, in-hospital mortality, or postacute facility discharge.

This study found that a prospective multicenter implementation of an electronic interprofessional-led discharge planning tool in a challenging and siloed environment was feasible. This is important, as many previous improvement studies were limited by complex interventions or cumbersome implementations, and it has been suggested that team-based care approaches would have limited success.^19,28,29,30^ Given the clinical setting with a heterogeneous patient population, this nationwide large-scale study, showing high tool use and systematic assessment of outcomes with no missing data, may fill an important gap in the current evidence about the effectiveness of interprofessional-led discharge planning to reduce LOS.

Second, given the fact that intention-to-treat analyses arguably better evaluate the effectiveness of interventions in clinical pragmatic studies, assessment of intervention fidelity is critical.^31^ Although we could not systematically assess whether the intervention was implemented as intended, the frequency of tool use in this study was approximately 80%, which is higher compared with other pragmatic studies.^32^ Nonetheless, a more comprehensive comparison with previous studies is largely limited, as many studies did not assess the adherence to intervention use and, thus, had an increased risk of rejecting potentially effective interventions that failed to work because they were poorly implemented or of accepting ineffective interventions that achieved desired effects caused by factors other than the intervention.^33^

Third, this bundled intervention was associated with a reduction in LOS of approximately 10.5 hours over 12 months, which was not seen in the control group. This is relevant not only for patients and clinicians but also for hospital authorities, as even a small decrease in LOS could unlock capacity for subsequent admissions in a hospital with a shortage of acute care beds. This is notable because previous interventions were not consistently associated with a decrease in LOS for patients with medical complexity.^19^ While our findings validate results from a previous meta-analysis^3^ showing that LOS was reduced for medical patients who were allocated to a discharge planning program, other investigations found no or even an increasing effect on LOS among unselected medical patients.^6,34^ Interventions in smaller and more selected patient populations, such as in those with heart failure, were more likely to be successful in reducing LOS.^35^ To increase reach, however, this study included a broad and heterogeneous population of medically complex patients with multiple acute and chronic morbidities. Fourth, the study intervention was not associated with safety concerns. This was also true for 30-day hospital readmissions, an outcome that was negatively affected by the SwissDRG implementation, the so far largest strategy in Switzerland to improve hospital efficiency.^36^ Previous hospital-based discharge planning interventions showed mixed results among medical patients. One systematic review^34^ found improved readmission rates while LOS increased among the intervention groups. Another systematic review^35^ found no difference in readmission, without, however, assessing LOS. In-hospital mortality and facility discharge, 2 balancing outcomes of this study, did not change in the intervention hospitals, supporting the safety of our intervention, and did not explain the reduction in LOS.

The large multicenter design, the heterogeneity in medical patients with multimorbidity, and the adaption of the intervention to local settings are all characteristics of a pragmatic clinical intervention, revealing the robust external validity of this study. We provided a standardized description of all interventions that can be adapted and transferred to different health care settings. The intervention’s adaptability was proven by this study, as local settings varied among the 7 intervention hospitals, even though 6 had the same EMR and some already had experience in research collaboration. We found that the implementation of In-HospiTOOL was associated with a significant reduction in LOS without an increase in hospital readmission and other balancing safety outcomes. Thus, these findings provide evidence of the potential effectiveness of interventions to safely reduce LOS and will also support hospital authorities and health care decision-makers in implementing interprofessional-led discharge planning interventions tailored to their local setting and patient spectrum.

### Areas of Further Research

Further research into the effectiveness of our intervention would be beneficial. To better evaluate the main factors associated with more efficient discharge planning, active comparisons may help to identify single elements of this bundled intervention that are useful to organizations. These could include experimental and nonexperimental comparative studies and look at the specific influence of potentially important moderators (eg, training methods, intensity of social worker rounding to detect postacute care demands early, monitoring strategies to evaluate intervention’s fidelity in terms of plausibility). Moreover, given the fact that there was still a progressive decline in LOS at the end of study period, the potential benefit of the tool might be underestimated. Therefore, potential sustainability of the intervention should be evaluated after the study intervention was stopped in most centers.

### Limitations

Our study has several limitations. First, although our findings are consistent with the implementation of the discharge planning tool, the nonrandomized design limits the ability to draw a causal link. However, it is unlikely that the changes in trend among the intervention group were solely caused by secular trends in the presence of a comparison group without such changes. Second, while the average LOS at baseline in this study was much longer than seen in many other countries’ hospitals, generalizability could be compromised. However, even in a setting of shorter LOS, the use of a structured interprofessional collaboration as an essential element in preserving safety of patient discharge seems justified. In addition, LOS might be overestimated in Switzerland by counting certain readmissions within 18 days of discharge as a single hospitalization; however, any resulting bias would affect the intervention and control groups similarly. Third, while the analyses were adjusted for several important measured confounders, unmeasured confounding, such as differences in socioeconomic status or practice variations between wards and hospitals, is likely and may have influenced outcomes. Fourth, the selection of intervention hospitals based on prior scientific collaboration may have introduced selection bias and could have reduced generalizability. Fifth, involved health care professionals, hospital authorities, and research assistants could not be blinded, which may have introduced reporting and participation bias favoring the intervention. Sixth, although the frequency of tool use was systematically assessed, we were not able to evaluate whether the tool was used in a plausible and intended way. Seventh, we did not perform a cost-effectiveness analysis nor an exploration on extra workload that both could be barriers for a broader tool use. Similarly, caregivers were not systematically interviewed to assess whether they perceived a change in their work satisfaction during the study. Furthermore, a certain risk of misclassification cannot be excluded given that administrative data were used; however, we do not expect that it influenced the outcomes, as both study groups would have been similarly affected.

## Conclusions

This nonrandomized controlled trial found that the implementation of an electronic interprofessional-led discharge planning tool was associated with a reduction in LOS among medical patients with multimorbidity without increasing the risk of hospital readmission, in-hospital mortality, or facility discharge. The findings of this study strongly support a broader implementation of embedded discharge planning programs to safely reduce LOS in unselected hospitalized patients with multiple medical conditions as routinely treated.

## Notes

Supplement 1.
Trial Protocol

Supplement 2. **eTable.** Crude Estimates of Length of Stay, 30-Day Hospital Readmission, In-Hospital Mortality, and Facility Discharge Among Intervention and Control Hospitals **eFigure 1.** Timeline and Study Design **eFigure 2.** Study Flow Diagram **eFigure 3.** Frequency of Tool Use Among Physicians and Nurses

Supplement 3.
Data Sharing Statement
