# Supplementary material for: Association of Interprofessional Discharge Planning Using an Electronic Health Record Tool With Hospital Length of Stay Among Patients with Multimorbidity: A Nonrandomized Controlled Trial
Source: JAMA Netw Open. 2022 Sep 28;5(9):e2233667. doi: 10.1001/jamanetworkopen.2022.33667 (PMC9520366; doi:10.1001/jamanetworkopen.2022.33667)
Supplement: Supplement 1. — Trial Protocol [file jamanetwopen-e2233667-s001.pdf]

Integrative Hospital Treatment in Older patients to benchmark and improve Outcome and Length of stay – the *In-HospiTOOL* study

A quasi-experimental, multicenter comparative effectiveness trial study protocol

Protocol version V1.1 (13.10.2016)

Principal Investigator and correspondence:

Prof. Dr. med. Beat Mueller, M.D.
Department of Internal Medicine
Department of Endocrinology, Diabetes and Clinical Nutrition
University Department of Internal Medicine,
Kantonsspital Aarau, Tellstrasse, CH-5001 Aarau, Switzerland
Email: happy.mueller@unibas.ch

Collaborations & research network

[1]Alexander Kutz, MD; [1]Daniel Koch; [1]Antoinette Conca; [1]Ciril Baechli; [1]Sebastian Haubitz; [1]Katharina Regez; [1]Ursula Schild; [1]Zeljka Caldara; [2]Fahim Ebrahimi, MD; [2]Stefano Bassetti, MD; [2]Jens Eckstein, MD PhD; [3]Juerg Beer, MD; [3]Michael Egloff, MD; [4]Vladimir Kaplan, MD; [5]Tobias Ehmann, MD; [6]Claus Hoess, MD; [7]Heinz Schaad, MD; [8]Ulrich Wagner; [9]Sabina de Geest; [1]Philipp Schuetz, MD, MPH, [1]Beat Mueller, MD

[1] Medical University Department, Division of General Internal and Emergency Medicine, Kantonsspital Aarau, Aarau, Switzerland;
[2] Division of Internal Medicine, University Hospital Basel, Basel, Switzerland;
[3] Internal Medicine Department, Kantonsspital Baden, Baden, Switzerland;
[4] Internal Medicine Department, Kreisspital Muri, Muri, Switzerland;
[5] Internal Medicine Department, Spital Zofingen, Zofingen, Switzerland;
[6] Internal Medicine Department, Kantonsspital Muensterlingen, Muensterlingen, Switzerland;
[7] Internal Medicine Department, Spital Interlaken, FMI, Interlaken, Switzerland;
[8] Swiss Federal Office for Statistics, Neuchâtel, Switzerland
[9] Department of Public Health of the Faculty of Medicine, University Hospital Basel, Switzerland

kutz.alexander@gmail.com; Daniel.koch@ksa.ch; Antoinette.conca@ksa.ch; ciril.baechli@ksa.ch; sebastian.haubitz@ksa.ch; Katharina.regez@ksa.ch; Ursula.schild@ksa.ch; Zeljka.caldara@ksa.ch; Fahim.ebrahimi@usb.ch; Stefano.bassetti@usb.ch; jens.eckstein@usb.ch; Juerg-Hans.Beer@ksb.ch; Michael.Egloff@ksb.ch; vladimir.kaplan@spital-muri.ch; Tobias.Ehmann@spitalzofingen.ch; claus.hoess@stgag.ch; heinz.schaad@spitalfmi.ch; ulrich.wagner@bfs.admin.ch; sabina.degeest@unibas.ch; schuetzph@gmail.com; happy.mueller@unibas.ch

Summary

Health care costs in Switzerland are high and rising also due to the aging, polymorbid population. In-hospital treatment is key contributor to the current cost explosions. In view of the expected demographic evolution, resource allocation becomes a national priority. There is a lack of evidence-based tools namely for elderly, polymorbid patients to improve the integrative inter-professional in-hospital care and the transition process in real-life of an emergency and acute care hospital setting. Also, there is no reference standard for quality benchmarking in Switzerland which is mandatory to compare quality of different institutions. To address these issues, we propose a pragmatic multicenter “before-and-after” trial to study the effect of an inter-professional inpatient management tool (“In-HospiTOOL”) on length of stay and other patient outcomes. This tool combining several patient discharge measures was developed at our institution in an intensive multi-professional collaboration for the past ten years. For external multicenter validation of this tool, we will prospectively include consecutive polymorbid medical patients upon admission to the medical ward. Because patient-level randomization is not feasible for an intervention that focuses on the process of care, we will use a quasi-experimental approach and compare outcomes before and after hospital-wide implementation of the management tool. We will use time-trend analysis to compare length of stay before and after tool implementation. Data from other Swiss hospitals from the Swiss Federal Office of Health (Bundesamt für Gesundheit, BAG) serve as a control population. We target the inclusion of 45`000 patients over an 18-month period in at least five Swiss hospitals. The trial will inform us whether the “In-HospiTOOL” improves inter-professional team work and thereby reduces length of stay without negatively impacting subjective and objective markers of patient outcomes. The large amount of patient data collected within this trial will enable comparison of transition processes within different hospitals and establish a benchmarking for patient care quality addressing all three *modules* outlined by the “National Research Program” (NRP) 74 call. Our trial synergizes funds, national networks and, thus, will likely become a milestone in the current public healthcare discussions.

1. Research

1.1 State of research in the field

A comprehensive in-hospital patient management with adequate and human resource allocation is arguably *the* major challenge of health-care systems, governments, and societies worldwide [1], especially in elderly, frail, polymorbid and less well educated, or cognitive impaired patients [2]. Improved diagnostic and therapeutic measures have increased life expectancy, yet, lead to a growing number of polymorbid, elderly patients. Chronic disease burden, co-morbidities, and frailty are key risk factors for emergency hospitalization. The trigger for hospitalization is often a *per se* minor acute disease (e.g. infection of the respiratory tract), which – on top - disrupts the fragile bio-psycho-social homeostasis of polymorbidity. Too often, post-acute discharge to a nursing care facility is allegedly inevitable. High demands for medical, nursing and social care put a strain on our health care resources [3, 4]. *The need for patient management tools to improve the transition process and allocation of health care resources in routine clinical care particularly for the inpatient setting is obvious.*

The Swiss health care system has some advantageous approaches in coordination between patients and physicians as compared to other countries [5]. There is, however, room for improvement, particularly in terms of accountability for quality, appropriateness, and costs of health care services [6]. Cantonal responsibility for planning and delivery of health care services, partial financing of hospitals, as well as provision of subsidies for insurance premiums makes a national assessment and steering challenging. Federal, cantonal, and private organizations require and publish benchmark data from hospitals. Recently, internet portals started to publish comparisons for performance of health care providers – although the reliability and representativeness of these comparisons can be questioned.

Many of these quality-mirroring tools are indeed misleading, as they do not reflect true resource need and use, especially in elderly, polymorbid patients. Also, there is no validated tool for optimization of patient flow and discharge process. Some hospitals have developed internal instruments with more or less sophistication and practicability. Safety, effectiveness, cost-efficiency, transferability and external validity of these tools are, however, understudied [7, 8]. Health care authorities and hospital executives lack scientific evidence to promote, enforce and sanction changes or to guide the flow of polymorbid patients. There is, thus, urge to validate benchmarks and inter-professional tools to improve patient management, flow and length of stay without compromising patient outcome and functional independence despite chronic polymorbidity in a pragmatic multicenter setting [9]. *Our comprehensive proposal targets all three modules of the “National Research Program” (NRP) 74.* This is outlined in more detail below.

Several approaches have addressed in-hospital resource allocation. Yet, a comprehensive patient management tool focusing on the overall in-hospital health care process in polymorbid patients and validated in a multicenter setting – as described in Module 1 of in this proposal - is still missing.

Mis-utilization and suboptimal resource allocation challenges safe and efficient, patient-centered in-hospital flow from the emergency department (ED), medical ward, and transition to home or post-acute care facilities [10]. Inadequate use of health care resources is by far more than technical procedural flaws [11]. Expected benefits of hospitalization must be weighed against clinical uncertainty, risks associated with inpatient environment [12], and costs associated with a hospital stay in the health care provider`s site of care decision [13]. Errors that lead to preventable deaths are more common in polymorbid patients than in other health care settings [14]. Because the majority of medical patients with chronical illness is hospitalized through the ED (non-electively), optimized resource use has to start at the ED with an improved triage. A nontrivial proportion of ED patients are - in retrospect - deemed non-urgent but, nevertheless, had procedures performed and were admitted to the hospital, including even intensive care units (ICU) as expression of over-treatment and resource misuse [15]. Timeliness of care is a key target and quality measure to prevent unfavorable outcome [16]. Excessive short-term unscheduled ED readmissions is another key measure of poor quality [17, 18]. Crowding is also associated with negative patient-relevant outcome, including poorer care, adverse events, medication errors and lower satisfaction [19, 20]. Lastly, post-acute care planning - integrated into an accurate risk stratification on admission [21] - has the potential to

reduce length of stay and prevent functional disability associated with prolonged hospitalization [22-24].
In the transition from hospital to home, many patients experience adverse drug events [25], have inadequate follow-up [26], and manifest difficulties with the execution of discharge instructions [27]. Transitional care has shown beneficial effects on readmission rates [28]. However, the effects of transitional care on mortality are inconsistent [29, 30], and there seems to be no long-term effect on activities of daily living [31]. Conversely, a reengineered discharge program decreased hospital utilization by implementing a nurse discharge advocate and a clinical pharmacist working together to coordinate hospital discharge, educate patients, and reconcile medications [32].
Conflicting data drove us to design an integrative patient management tool, especially focusing on older chronically ill in-hospital patients while being acutely sick (Module 2).
The optimal organization of routine medical ward care in mostly polymorbid, elderly patients of general internal medicine received less attention than the management of specific diseases. Specifically, there is a lack of large studies focusing on polymorbid patients and no improvement of objective patient outcomes [33]. The inter-professional team care approach with a comprehensive geriatric in-hospital assessment has been found effective to increase patients' likelihood of being alive and in their own homes after an emergency admission to hospital [34]. Conversely, many prior studies have been unable to link interdisciplinary team care interventions to change in existing metrics, partly because of limitations in methodology and outcome measures [35].

Recently, innovative concepts to synergize the concepts of implementation science, precision medicine, and learning health care systems have been advocated [36]. Using this experience, we integrate evidence-based strategies (e.g., system change interventions, training, supervision, quality monitoring tools) into real-world practice [37] (Module 3).
Health care providers and payers spend substantial resources in collecting, analyzing, and reporting data on health care service performance [38]. Beyond the issue of high diversity and lack of validation of these measures, there is an ongoing dispute which performance data optimally reflect high quality of care. Some metrics capture health outcomes or processes that have major effects on overall health, but others focus on activities that may have minimal effects [38]. *The lack of consensus regarding performance benchmarking data in Switzerland is a major obstacle for quality improvements* [39-42].
For an optimal translation into clinical practice, availability and accessibility of high-quality data is a prerequisite, including accessibility for patients [17]. Several initiatives have proposed to share data with the public [43]. Availability of anonymized patient-level data from clinical trials can permit verification of original results, enhancing public trust and accountability, facilitate other critical research (e.g., evaluation of adverse event rates according to compound class or subpopulation or identification of surrogate end points), and avert duplicate trials [44]. "Data dumpsters" must be prevented, i.e., simply making more data openly available without linking them to relevant documentation and analyses that are applied to improve health [45].

1.2 Personal contribution to research in the field

For more than 15 years and supported by the Swiss National Science Foundation (SNF), our inter-professional, multicenter research group published studies investigating strategies for improving management of chronic, polymorbid medical patients. Below we summarize our track record specific to the different *modules* outlined in the NRP 74 call.

Module 1: Resource (mis-)utilization & allocation

We have optimized resource use in the fields of emergency triage, antibiotic stewardship and malnutrition. Also, a main priority of our research was to optimize site of care decisions in the ED and reduce length of stay in the inpatient setting. In a secondary analysis of a Swiss-wide multicenter trial on antibiotic stewardship in respiratory tract infections (SNF 3200B0-116177, ProHOSP) [46], we found that even before 2012 Swiss hospitals with DRG based financing had a 20% shorter length of stay as compared to fee-for-service (FFS) hospitals without apparent harmful effects on patient outcomes, satisfaction and quality of life [47]. When looking at barriers for early discharge, independent of type and severity of disease, misperceived high severity and expected mortality were predominant reasons why treating physicians, nurses, patients and their relatives believed that inpatient management was necessary [48, 49]. We also reviewed psychological distress in medical patients seeking emergency care for somatic

reasons [50]. Again supported by the SNF (32003B_135222, OPTIMA II study) and the Canton of Aargau (Departement für Gesundheit und Soziales, DGS AG), we validated an inter-professional risk assessment tool including clinical and biochemical parameters for an improved risk estimation in polymorbid patients with respiratory infections to safely increase the outpatient treatment rate and to reduce length of stay [51, 52]. This research was undertaken in an inter-professional team of nurses, physicians and social care workers often in a multicenter setting, and including and addressing needs of patients and their relatives.

Module 2: Inter-professional collaboration, integral hospital and post-acute patient flow

After revealing inter-professional barriers for earlier patient discharge in patients with respiratory infections [48, 49, 53], we focused on more heterogeneous, polymorbid medical inpatients [54-59]. We validated the post-acute care discharge (PACD) score [21] as a highly predictive nursing risk assessment tool to predict post-acute institutional care thereby allowing early involvement of social workers to facilitate transition [60, 61]. Similar results were also found in a very recent large prospective cohort study, investigating >1800 medical and neurological patients (Conca A., et al., submitted). Later, we completed a randomized intervention study to investigate the effects of intensified social worker integration (Prins M., et al., submitted). We implemented successfully a Nurse Led Care (NLC) concept for medically stabilized patients with high nursing care needs [62-64], to achieve a better functional status, a higher psychological well-being, and a lower unplanned readmission rate [63, 65, 66], [Conca A. et al., poster award 3rd prize, SAMW, 2015, Berne].
Using our inter-professional expertise and up-to-date electronic medical chart technology, we developed an inter-professional patient management tool ("Visitentool", **Figure 1** and **3**) [Conca A. et al., poster presentation, CareART, 2014, Basel; Conca A. et al., poster presentation, Gesundheitssymposium, 2014, St. Gallen]. This platform includes information from (a) the initial patient assessment to improve decision regarding inpatient vs. outpatient care and for early prediction of post-acute care needs ("Ersterfassung" including PACD score) and (b) daily patient-assessments on the ward to improve decisions regarding early patient discharge for safe transitions from hospital to home or to a post-acute care institution [67]. Using this platform, physicians, nurses and social workers – of course, adapted to needs and wishes of patients and relatives, respectively - communicate discharge-relevant information daily using a simple, intuitive color code, including estimated date of discharge from point of view of each profession.
A comprehensive discharge instruction program including patient education and teach-back methodology [68] about relevant diagnoses and medication, instruction about follow-up procedure with coordination of appointments (physicians, nursing home) and clarification of logistic details (transport, location) is used for all patients [32], [Kutz A. et al., poster award 1st prize, SAMW, 2014, Berne; Kutz A. et al., Swiss Quality Award meeting, 2014, Solothurn].
The benefits of our inter-professional efforts became evident in a sub-analysis of the recent STEP-Study (SNF-Professorship to Prof. Mirjam Christ-Crain). After adjusting for disease severity in patients with pneumonia, the Kantonsspital Aarau had an adjusted 3-day shorter mean length of stay as compared to other Swiss cantonal and university hospitals with similar patient outcomes [69].

Module 3: Benchmarking to advice health care authorities and stakeholders

Supported by a grant from the "Swiss Academy of Medical Sciences" (SAMS/SAMW), we widened our monocentric focus on risk assessment, patient flow and benchmarking in the multicenter "Triage Study" including more than 7`000 patients in Switzerland, France and the United States [67, 70]. We found clinical parameters and blood markers from distinct pathophysiological pathways to be helpful for early risk assessment in a heterogeneous group of polymorbid medical inpatients independent of underlying diagnosis. We also validated clinical triage scores such as the Manchester triage system (MTS) [71] for estimating patient acuity [72]. Very recently, we defined predictors for delayed ED care in international medical polymorbid patients with acute infections [73], and a larger comparative quality measurement involving ~3000 patients from different medical disciplines revealed similar results, supporting the concept that further benchmarking improves health care service (Burgemeister et al., submitted). We have established an electronical monitoring and reporting system, enabling clinical user oriented benchmarking ("Nutzerorientierte Kennzahlen, NOK", **Figure 2**) to monitor hospital processes, delays in hospital transition and barriers for discharge stratified by profession (i.e., physicians, nurses, social workers) [51, 70, 71]. For this purpose, we monitor patient outcome and satisfaction by telephone interviews 30 days after admission with ~15'000

patient interviews per year being done at our hospital with an exceptionally high follow-up rate of >90%. To date, we gathered data of >30`000 inpatients in a large observational database (OPTIMA-TRIAGE). Based on this dataset, several analyses have been published regarding outcomes of medical inpatients [50, 70, 73-76] [Kutz A. et al., poster award 2nd prize, 7th Symposium of the Swiss Clinical Trial Organisation, 2016, Lausanne]. We regularly report key measures of health care and patient outcomes to the hospital governing board and cantonal authorities. We are actively involved in the MIVAG-network (“Masterplan Integrierte Versorgung Aargau”), a pioneering cantonal initiative for integral collaboration of pre-, peri-, post-acute and chronic health care.

1.3 Detailed research plan

Research question and specific aims

We propose a large Swiss-wide trial to investigate the effects of a patient in-hospital management tool (“In-HospiTOOL”) successfully validated in a single center setting of a Medical University Clinic. For this multi-center validation, we will use a “before-and-after” design and an interrupted time series (ITS) statistical approach. Nested in this main trial, we will gather detailed treatment and outcome data of mainly elderly, polymorbid medical patients during the in-hospital stay and 30 days after admission to investigate differences in resource use (*Module 1*), inter-professional collaborations (*Module 2*), and to establish representative benchmarking data to promote measurement and display quality of care data [77] across different Swiss hospitals (*Module 3*). Thus, as outlined in detail below, we are addressing and synergizing all three *modules* of the NRP 74 call with this proposed study. This maximizes efficiency of our comprehensive effectiveness research project “In-HospiTOOL” and reduces costs.

Module 1: In a pragmatic Swiss multicenter study enrolling ~45’000 elderly polymorbid medical patients we will study the effects of the multi-professional “In-HospiTOOL” (**Figure 1**) on length of stay, our primary endpoint. As secondary endpoints we will explore reasons for delays during emergency treatment, in-hospital patient flow, transition to post-acute care, and readmissions (synergies of *Module 1* and *2*). The elements of the “In-HospiTOOL” were developed at the Medical University Clinic of the Kantonsspital Aarau in an inter-professional effort to optimize inter-professionalism and early safe discharge of patients. This tool is now ready to be externally validated.

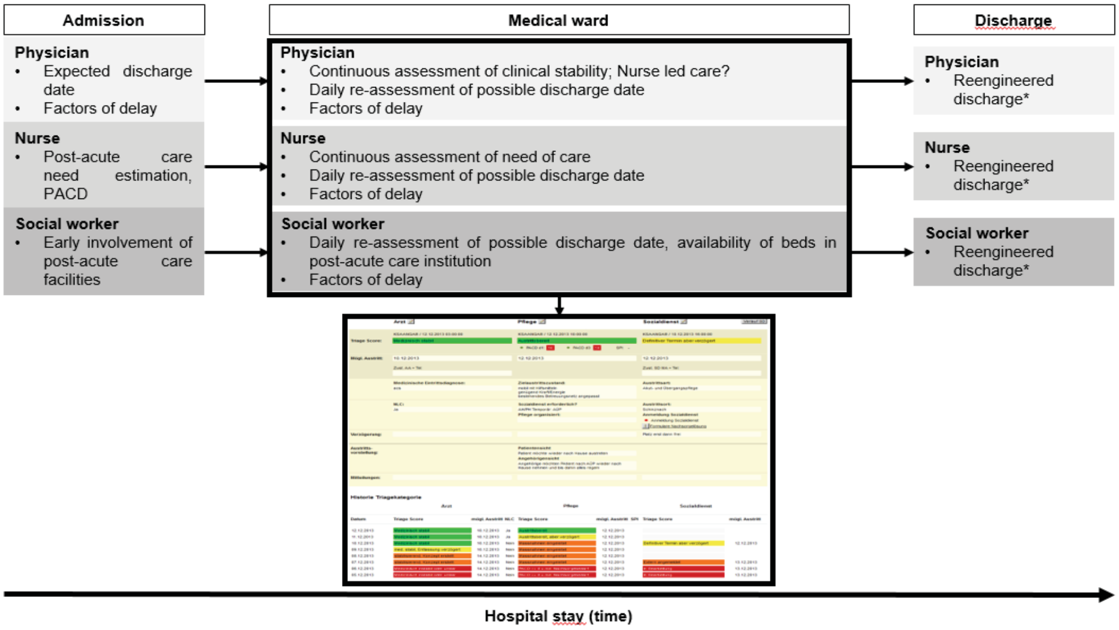

*11-item checklist including patient information regarding medical diagnoses, expected risk and prognosis, medication, coordination of follow-up appointments (physicians, nursing home), clarification of logistic details (transport, location).

Figure 1. The “In-HospiTOOL”. An integrative patient management tool. The “In-HospiTOOL” has three components involving admission (inter-professional initial assessment, *“Ersterfassung”*), medical ward (inter-professional daily re-assessment, *“Visitentool”*), and discharge (inter-professional patient education, reengineered discharge [32]). PACD, Post-Acute Care Discharge [21].

Module 2: Using the data gathered in the main trial (*Module 1*), we will identify differences in inter-professional collaboration and barriers across participating hospitals. We will analyze factors for delay (i.e., pending diagnostics, medical treatments, administrative and organizational elements), effective time to hospital discharge after involving external institutions (time from transfer application to transfer), and satisfaction of patients. This allows cross-sectional and longitudinal observation of polymorbid patient transition and may help to realize an improved health care continuum from initial presentation to the final disposition. This will improve our understanding of factors that influence the transition of care from acute to post-acute care institutions.

Module 3: By focusing on outcome data and using a "Delphi approach" within an inter-professional sounding board, we will establish a patient risk-adjusted health care monitoring system to benchmark patient outcome and satisfaction data. This "Cockpit" approach (**Figure 2**) supports better comparability of internal quality measures among hospitals in Switzerland. At the same time, it provides objective and transparent quality data for future display to patients and policy makers. The sounding board will include representatives of all health care professions in ambulatory, hospital and post-acute care, insurances, public health, health economics and statistics, ethical boards, as well as hospital administrators, cantonal and federal authorities (a detailed sounding board constitution is described below in section 2.3 - *Implementation partners*). We also plan to disseminate these findings to the public (see section 2 - *Implementation*).

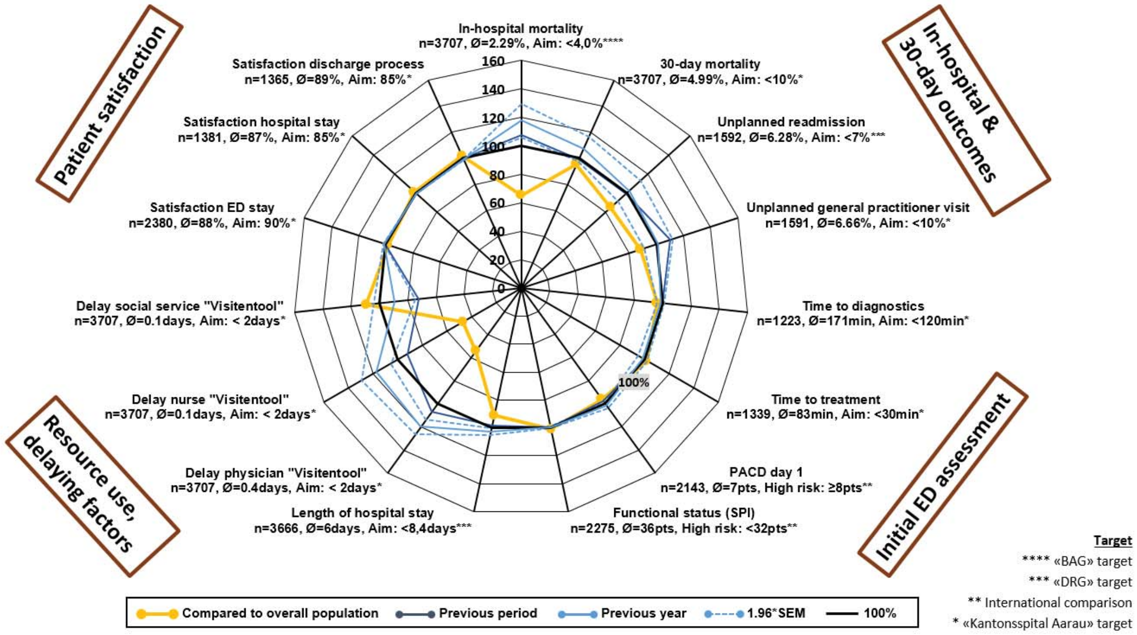

Figure 2. The "Cockpit". For clinical user oriented benchmarking (Nutzerorientierte Kennzahlen) based on patient data from our own database including >30`000 medical patients. Quality data from different dimensions is displayed comparing different time periods. We report data about in-hospital and 30-day outcomes (i.e. in-hospital and 30-day mortality, unplanned readmissions or general practitioner / ED visits), initial ED assessment (i.e. PACD score, ED procedure delaying factors), resource use and delaying factors (i.e. ED and medical ward delaying factors, length of stay), and patient satisfaction (i.e. satisfaction with ED, ward, and discharge process).

Theoretical aspects, hypotheses

There is a lack of evidence-based tools for management of polymorbid medical patients throughout the in-hospital stay with transition to post-acute care institutions. We propose a trial that will close this gap by studying the effects of the "In-HospiTOOL" on resource use including length of stay, inter-professional collaboration, and at the same time will give transparent information on outcome data and barriers to transition across several Swiss hospitals.

Module 1: We hypothesize that implementing the "In-HospiTOOL" in a nationwide multicenter setting will significantly shorten length of stay without compromising patient outcomes and functional independence. Tight inter-professional collaboration enabled through an electronical communication platform ("Visitentool", **Figure 3**) and identification of the delaying factors in the patient flow will result in decreased waiting times contributing to the shortening of length of stay.

Module 2: Transparent inter-professional communication will reveal factors for delay in these polymorbid patients (pending diagnostics, medical treatments, administrative and organizational elements) throughout the hospital stay. Doing so, we will identify regional and socioeconomic (e.g., health care insurance status) differences in the patient continuum. We hypothesize that longitudinal observation of patient flow will further allow us to measure effective time from initial request to a post-acute care institution to effective transfer with corresponding internal and external delaying factors. We will systematically examine patient satisfaction and – based on our own research - hypothesize no reduction of it. Also, we will investigate reasons for low satisfaction.

Module 3: The buildup of a large dataset including comprehensive patient information (demographics, clinical, organizational, health insurance status) will be a basis for future data sharing in Switzerland [78]. We hypothesize that this dataset from several Swiss hospitals will allow identifying associations of management factors and outcome data, thereby helping us to better understand how interventions affect patient outcomes. Convocation of a multi-professional sounding board with tailored implementation interventions [79] will be inevitable for built-up a data warehouse and thus, broad dissemination of our results which has potential to improve health care service.

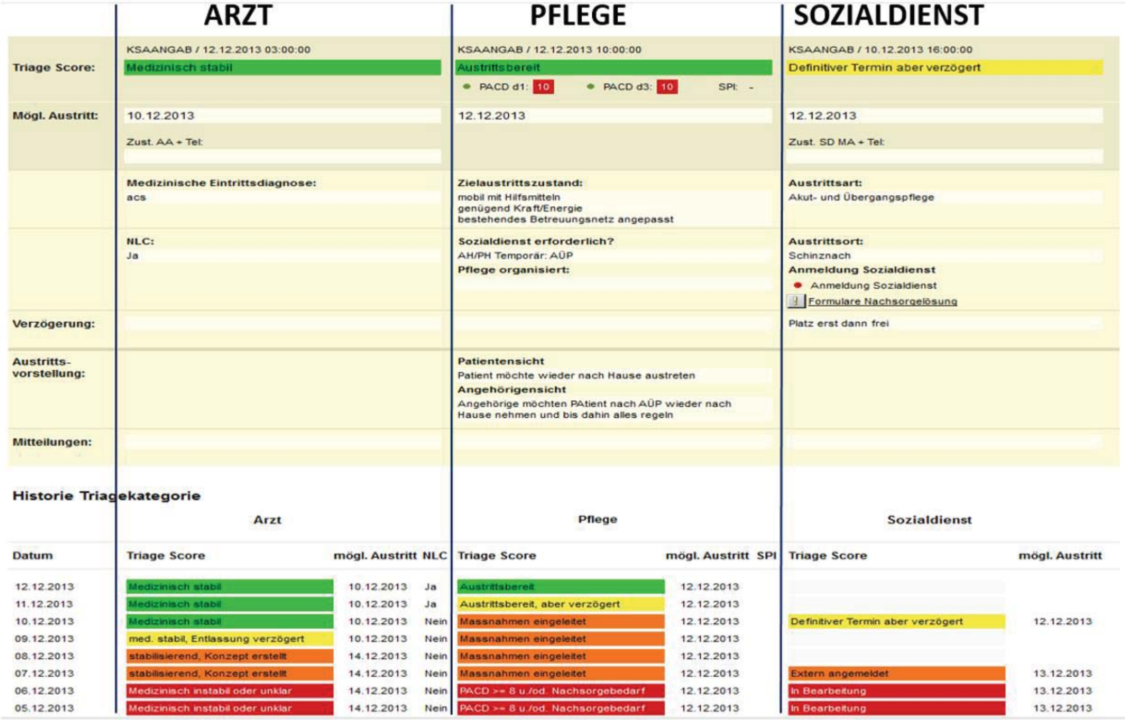

Figure 3. The "Visitentool" (german). Inter-professional collaboration via an electronical communication platform. Nursing and physician staff as well as social services daily assess the clinical and functional situation about possible discharge (using simple, intuitive color coding) and propose possible discharge dates. Also, reasons for delays in discharge are being monitored. For medically stabilized patients with high nursing care needs we institute a Nurse Led Care (NLC) concept.

Achievement of specific aims

Intervention

In five secondary and tertiary care hospitals we will prospectively include consecutive medical patients upon admission to the medical ward. The main intervention will be the hospital-wide implementation of the “In-HospiTOOL” with its different components for discharge management.

In a planning and pilot phase (see section 1.4 – *Timetable and milestones*), study site investigators and staff (physicians, nurses, social service, clinical nurse scientists, information technology representative) will meet to define a basic orientation program to educate involved study personnel and hospital collaborators about the intervention. We will teach staff how to adhere to electronic ED, medical ward, and discharge assessment and to empower patients and families to attend post-discharge follow-up appointments, manage medications, and identify and manage symptoms using teach-back methodology [68]. We plan “learning sessions” in a 6-month interval to troubleshoot and manage issues with the intervention program.

In detail, upon ED admission, we will perform two distinct triage assessments regarding medical and nursing risk. Physicians will decide about initial site of care (need for in-hospital treatment *versus* outpatient treatment) and estimate the possible discharge date as a basis for further inter-professional daily medical ward re-assessments. ED nurses will determine the PACD score for estimating the need for post-acute care transition to a post-acute care institution, enabling early involvement of social workers in high-risk patients. Physicians will systematically collect delaying factors of the ED process.

For medical ward patients, we will daily re-assess inter-professionally patient discharge management using the “Visitentool” (**Figure 3**). Physicians, nurses and social workers enter/modify the expected discharge date as well as information regarding clinical and functional stability, and organizational status (using a color code). Entering of factors responsible for delays in patient flow will be part of the assessment.

As a previously published *reengineered discharge* tool has provided significant improvement in hospital utilization after discharge [32], we have incorporate this tool in the “In-HospiTOOL” discharge process. Herein, physicians, nurses, and social service will work with patients during their hospital stay to arrange follow-up appointments, confirm medication reconciliation, and conduct patient education.

We will perform telephone interviews with all patients 30 days after hospital admission to assess their satisfaction and outcome data. Other than that, patients will receive routine hospital care without interference by the study team.

Intermediate data

Module 1: In a first 6-month observational phase, we will generate baseline data of ~15`000 medical patients from five representative Swiss hospitals. The data will include length of stay (primary endpoint) and data about patient and disease characteristics, demographics, routine process variables, and outcome data obtained after 30 days with structured telephone interviews. The data set will include information about the in-hospital process including relevant diagnostic and therapeutic interventions as well as short-term post-acute care period. During the 6-month of implementation, again ~15`000 patient data regarding length of stay and a reduced number of pertinent baseline data will be gathered. After implementation of the “In-HospiTOOL” we will generate another ~15`000 interventional patient data similar to the initial observation phase. This approach has the advantage to distinguish effects of the “In-HospiTOOL” from a difference in secular trends in the controls and intervention group. With this “before-and-after” design we will gain insights about the effect of “In-HospiTOOL” on resource use in term of length of stay (primary endpoint), delays during emergency treatment, in-hospital patient flow, transition to post-acute care, and readmissions (secondary endpoints). This will be the basis for further monitoring of resource use.

Module 2: For *Module* 2, in-hospital and outcome data gathered in *Module 1* will be used, but analyzed differently. A 6-month observational phase will provide cross-sectional data, allowing inter-hospital analyses of patient management. Specifically, in the intervention phase, we will focus on the effect of multi-professional collaboration on patient management and flow. We will gain data about delaying factors (pending diagnostics, medical treatments, administrative and organizational elements), effective time to hospital discharge after involving external institutions (time from transfer application to transfer), and satisfaction of patients. Using data from structured telephone interviews, we will also include information about the functional status of these polymorbid patients.

Module 3: We will focus on outcome data gathered during the in-hospital stay and the 30-day interview in the main trial. Using the "Cockpit" approach (**Figure 2**), we will propose standardized benchmarking data for future evaluation of quality of care. Main measured elements will be: in-hospital and 30-day mortality, unplanned readmissions or general practitioner / ED visits (in-hospital and 30-day outcomes), PACD score, ED procedure delaying factors (initial ED assessment), medical ward delaying factors, length of stay (resource use, delays), and satisfaction with ED, ward, and discharge process (patient satisfaction). Collection of this comprehensive outcome data will define novel and validate currently used quality measurements which was previously been noted as a major challenge [41].

Methods

Setting and study design

This is a prospective Swiss-wide "before-and-after" trial investigating the effects of a new patient in-hospital management tool ("In-HospiTOOL") on length of stay and other outcomes using two complementary, quasi-experimental analyses: difference in differences and an interrupted time series (ITS) (*Module 1*). Nested in this multicenter comparative effectiveness health care research trial, we will gather detailed treatment and outcome data of polymorbid medical patients during the in-hospital stay and after 30 days to investigate differences in resource use, inter-professional collaborations (*Module 2*) and to establish representative benchmarking data to promote measurement and display of quality of care data across Swiss hospitals (*Module 3*).

As of July 2016, the following hospitals have agreed to participate medical department-wide in the "In-HospiTOOL" study: University Hospital Basel, Kantonsspital Aarau, Kantonsspital St. Gallen, Luzerner Kantonsspital, Kantonsspital Fribourg. This allows us to collect representative national-wide patient-centered data from polymorbid patients. All senior executive leaders have reassured full support for an optimal implementation of the "In-HospiTOOL" in their hospitals.

Data collection process

The study period is divided into a 6-month observational period, a 6-month implementation period followed by a season-matched 6-month intervention period. The period of "In-HospiTOOL" implementation in the participating hospitals will be devoted to technical implementation, training of involved study personnel and physicians, and pilot testing. We will collect data throughout all three study periods by using electronic medical records and will contact all patients 30 days after hospital admission by phone interview.

Endpoints

The primary endpoint of this study is length of stay within 30 days after admission including readmissions during this period (corresponding to *Module 1*). Length of stay will be verified based on hospital data for the index hospital stay and complemented by 30-day interviews regarding possible secondary hospitalizations. As described in the statistical plan, we will use shared data from the Federal Office of Public Health (FOPH, BAG) about length of stay and a reduced set of basic patient information (i.e., main disease based on DRG code, main comorbidities, age, gender, health care insurance, home of residence). To grant access of BAG data we contacted the "Bundesamt für Statistik, BFS" at the end of June 2016. Preparation to close a data protection contract is ongoing.

Secondary endpoints (corresponding to *Module 1 - 3*) include measures of patient-centered outcomes (i.e., in-hospital and 30-day all-cause mortality, unplanned readmissions or unplanned general practitioner / ED visits, delaying factors of ED- and medical ward`s flow, institutionalization, effective time to hospital discharge after involving external institutions (time from transfer application to transfer), satisfaction with ED, ward, and discharge process, functional status (incl. quality of life), and overall hospital costs).

To study hospital internal processes and effect of inter-professionalism, we will look at compliance and agreement of the three health professions (physicians, nursing, and social workers) in the use of "In-HospiTOOL", and delays from the anticipated to the effective discharge date as compared to discharge date anticipated by the different health care professionals on admission and during the course of the hospital stay. We will use the above mentioned outcome data set as benchmark to establish a risk-adjusted resource and quality cockpit to compare different hospitals and demographics (corresponding to *Module 3*).

Independent variables

The primary exposure variable of interest is the intervention, i.e. the implementation of the "In-HospiTOOL". As outlined in the statistical plan, we will adjust our model to the following covariates: demographics (age, gender, health care insurance, home of residence [home versus facility]), main diagnosis (grouped using the "14 - International Classification of Diseases (ICD-10)" [80]), comorbidities (using the Elixhauser comorbidity index [81]), and study center.

Statistical analysis and sample size

Module 1: Because patient-level randomization is not feasible for an intervention that focuses on the process of care and differences in the patient population may occur due to epidemiological variations, we will assess the effects of introducing the "In-HospiTOOL" using two complementary, quasi-experimental analyses. This statistical approach was recently used successfully by one of our collaborators (Prof. E. J. Orav, Harvard School of Public Health, USA, see **Figure 4**) [82].
We will adjust outcome analyses in the difference in differences and interrupted time series (ITS) model for important covariates such as age, gender, health care insurance, home of residence, main diagnosis, comorbidities, and study center. In regard to sample size considerations, we will include consecutive patients in each hospital over a 6-month period for the observation, implementation, and the intervention period. Given the large number of patients per clinic seen in routine (i.e. between 4`000 and 8`000) per year, we estimate to enroll approximately ~45'000 patients over 18 month of recruitment (6-month observation, implementation, and intervention period, each). This large amount of patient data will provide strong power to look into the effect of introducing a patient care tool in the overall medical hospitalized patient population and allow for subgroup analyses, as well as important post-hoc analyses.

<u>*Difference in differences*</u>

To determine whether there will be an overall effect on length of stay after implementing the "In-HospiTOOL", we estimate a patient-level logistic regression model. It will include lengths of hospital stay of "BAG" hospitals, all risk adjusters listed above, a variable for elapsed weeks to account for secular trends, and indicators for intervention period, intervention hospitals, and their interaction. The dependent variable will be length of stay (days in hospital) within 30 days. By testing for an interaction between intervention period and intervention population, we will assess whether there is a difference in the change in length of stay over time between the two control and the intervention populations (difference in differences). The difference in differences design does not require that the control and intervention groups have similar baseline characteristics but rather assumes that both groups would have experienced similar changes in outcomes over time without the interventional program.

<u>*Interrupted time series (ITS)*</u>

We will analyze the trends in length of stay from start of observation through the end of intervention period (18 months). For this purpose, we will conduct an interrupted time series (ITS) as a sensitivity analysis. We will implement the interrupted time series (ITS) using generalized estimating equations (GEE), to examine linear trends in weekly, hospital-level, risk-adjusted length of stay. We will analyze the change in trend between all three time periods. This approach has the advantage to distinguish an effect of the intervention from a difference in underlying secular trends in the control and intervention

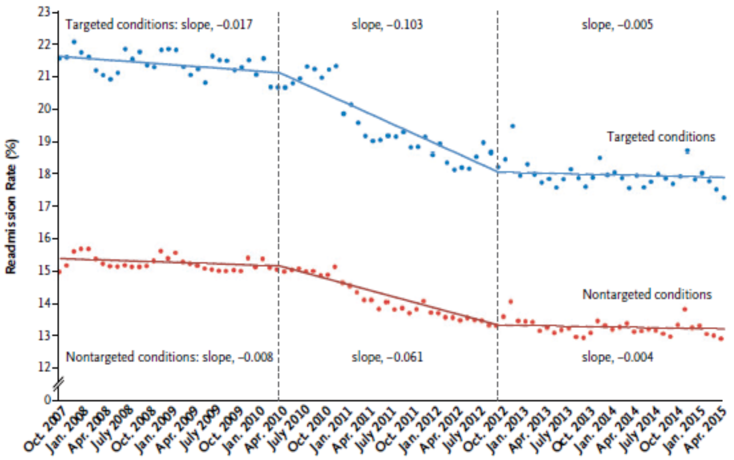

Figure 4. Interrupted time series (ITS) model. Schematic example showing readmission rates before (observation phase), during (implementation phase), and after (intervention phase) introduction of an intervention program.

populations (which could produce a misleadingly significant difference-in-differences result) and can also help to determine whether the intervention effects will sustain. We will calculate weekly-adjusted length of stay using linear GEE that includes all lengths of stay from the control and intervention populations and all above mentioned risk variables. This model also includes indicators for each week and interactions of these with an indicator for the control population. We will center all risk factors on their overall means and suppress the intercept to avoid omitting any month indicators. We will graph weekly length of stay for all populations over time and use the estimated weekly rates for control and intervention populations to calculate a weekly difference between the two populations. We then determine whether there is any overall decrease in adjusted weekly length of stay in the intervention period and whether there will be a time trend effect caused by the interventional program [83]. Because of suspected evidence of non-stationarity data we will use an autoregressive integrated moving average (ARIMA) model, with a 6-month autoregressive term to predict future impact of implementing the "In-HospiTOOL".
To summarize, we will use four statistically hypothesis tests during each period: First, are there significant trends in length of stay change during the period? Second, will the trend differ between the control and intervention populations (the interaction between time and control or intervention conditions) during the period? Third, will the trend during the intervention period differ from the trend during the observation period within all three conditions? Fourth, will the magnitude of the change in trend between the intervention and the observation period differ between the three conditions (the interaction between the change in slope and intervention or control conditions)? We will also use this models and tests for above mentioned secondary outcomes.

Module 2: To analyze regional and socioeconomic variations in quality of the transition process, we will cross-sectionally compare the patient management among participating hospitals. This qualitative part will give a summary of parameters that are relevant for the diagnostic, therapeutic, and discharge process (e.g., reasons for delayed hospital discharge from each professions` perspective). Also, we will focus on regional differences in disposability of post-acute care facilities and investigate associations with time to effective hospital discharge and length of stay using regression analysis as appropriate. Patient`s satisfaction in terms of the whole transition process will be another essential focus of investigation. To investigate trends over time, we will longitudinally analyze the impact of the "In-HospiTOOL" implementation. Depending on our available resources, we will develop a protocol to further investigate differences in costs among hospitals in a separate cost analysis (a detailed analysis plan will be developed).

Module 3: Using a Delphi approach we will invite a group of selected health care authorities (see section 2.3 - *Implementation partners*) to define further measures similar to **Figure 2**. We will study confounding factors for these quality outcomes for which analyses need to be adjusted to allow fair comparisons between hospitals. We will display data adjusted for confounders in a quantitative manner stratified according to time point and patient population. This will enable to monitor quality alterations and performance metrics over the study time as well as in the long-term process. Based on this benchmarking, again together with stakeholders, in a second study period we will define sustainable strategies for wider implementation and dissemination of study results and the "In-HospiTOOL" *per se*.

For all analyses, significance will be based on 95% confidence intervals. Data management and analyses will be performed with STATA statistical software (StataCorp).

Target population

Intervention population

To reflect "daily practice", we will include consecutive adult medical inpatients independent of their diagnosis during the observation, implementation, and intervention period into the analysis - similar to an intention-to-treat approach. Except for non-medical and non-adult patients there will be no exclusion criteria. As a quality control study with an intervention focusing on the hospital level rather than the individual patient level, we will ask the ethical review boards for a waiver of individual patient informed consent. This was granted for a similar monocentric pilot study in Aarau (EK 2012/059).

Control population

For our statistical approach as outlined above, we request data from the "BAG" to provide a nationwide comparability. We will use data on length of stay (primary endpoint) and age, gender, health care insurance, home of residence, main diagnosis, comorbidities, and study center for adjustment.

Expected results

As illustrated in **Figure 5**, we expect an inclusion rate of 15`000 patients in the intervention population for all three study periods (observation, implementation, intervention), each (total study population n~45`000). Based on our monocentric experience we expect the "In-HospiTOOL" to have a strong effect on the inter-professional team work in this polymorbid setting which results in reduction in length of stay of at least 1 day [69]. We also expect that patient outcomes are not negatively affected by the intervention with no increase in ICU admission, mortality, unplanned readmission, unplanned general practitioner visits and low satisfaction. A safe reduction of length of stay will have positive implication on overall hospital costs.

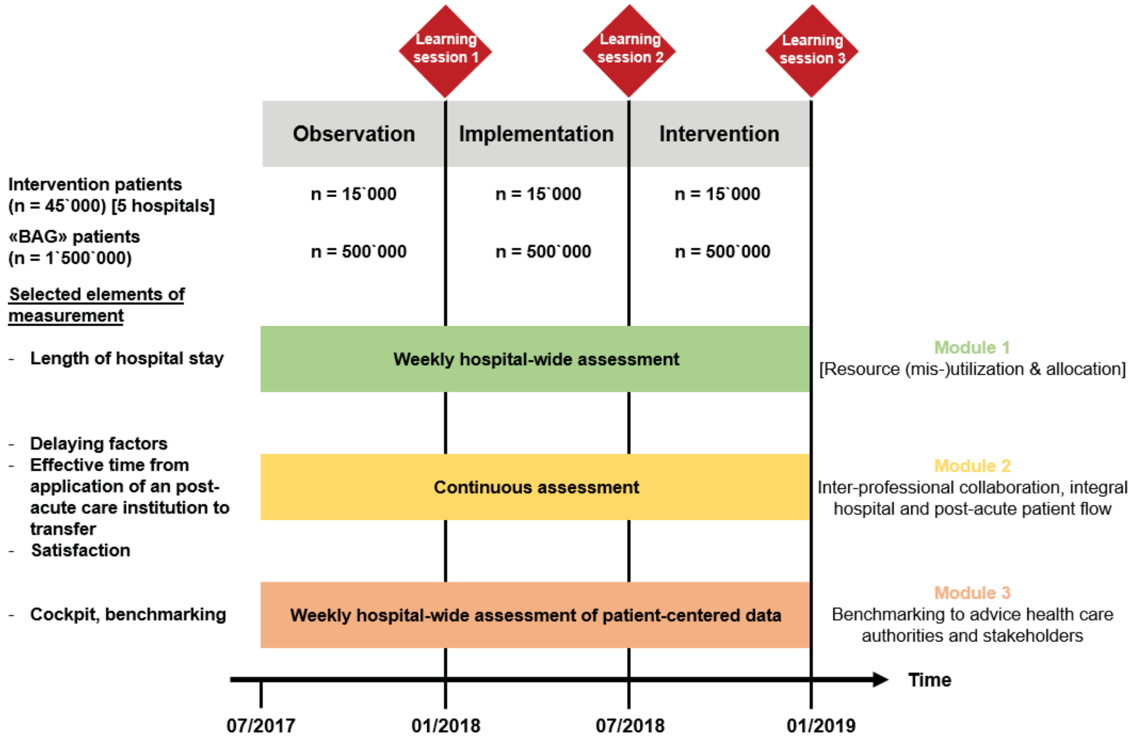

Figure 5. Main study timeline. Endpoint data will be collected throughout 18 months during the observation, implementation, and intervention period, and will be weekly reported. Education will be provided during the implementation and intervention period. Learning sessions are thought to compile intermediate data and to improve implementation and intervention processes. "BAG", Bundesamt für Gesundheit.

1.4 Timetable and milestones (Management Plan)

Our study addresses and synergizes research question in all three *modules*. We have already involved all leading authorities of the above mentioned hospitals and will organize an investigator meeting upon permission to start the study. For the intervention trial, we will do a 6-month planning and pilot-phase starting in January 1, 2017. This will be followed by a 6-month observational period with patient-centered data generation and 30-day follow-up phone interviews. The 6-month implementation period from January 1, 2018, through June 30, 2018 will be largely devoted to the technical implementation of the "In-HospiTOOL" in all involved interventional hospitals, training of involved personnel, tight monitoring by study nurses, and 30-day follow-up phone interviews. The intervention period will take place from July 1, 2018, through December 31, 2018. Thereafter, we have dedicated 18-month to complete the 30-day follow-up of all patients, and finish all endpoints of the *modules 1-3* as well as ancillary projects.

**Objectives**	Jan-Jun 2017	Jul-Dec 2017	Jan-Jun 2018	Jul-Dec 2018	Jan-Jun 2019	Jul-Dec 2019	Jan-Jun 2020	Jul-Dec 2020
**Main Study**								
Ethical approval, investigator meeting (2x), planning & pilot phase	■		■					
Scientific review, incl. publication	■							
Observational phase, active patient enrollment		■						
Implementation phase, active patient enrollment			■					
Intervention phase, active patient enrollment				■				
Finishing follow-up, database finalization					■			
Data analysis and manuscript preparation				■	■	■		
Manuscript publication						■		
**Secondary analyses as preparation for national-wide dissemination**								
Data analyses					■	■	■	■
Manuscript preparation and publication							■	■
**Administration**								
Benchmarking, monitoring, process optimizing		■	■	■	■	■	■	■
**Dissemination, national-wide implementation**								
Sounding board meeting, preparation*						■	■	■
**Requested funding period (4 years)**	■	■	■	■	■	■	■	■

Table 1. Schedule and milestones of the In-HospiTOOL-Study; *Details and milestones of the dissemination process are outlined in section 2. (*Implementation*).

2. Implementation

There is a gap between innovations in health care research and their implementation in routine practice [84]. Four key factors ensure that “In-HospiTOOL” is an important project for health authorities, hospital administrators, health care professionals, and patients to benefit from scientific advances with sustained effects for routine clinical care [36]. First, we propose a pragmatic comparative effectiveness trial involving major Swiss hospitals in which a number of management tools will be tested in clinical practice (“real-life”) in consecutive patients addressing patient-relevant, subjective and objective outcome parameters [85]. Second, implementation of the “In-HospiTOOL” will involve all key players in each hospital from nursing and social worker as well as physician staff and hospital administration. Third, the study requires permanent adaption of the electronic health record systems. Assuming a positive effect of this tool concerning resource use, we expect a high motivation of other institutions to adapt their processes in a similar way. Fourth, we will publish results giving details about the specific items included in the “In-HospiTOOL” not only as research papers but also selected content on classical news media (interviews with newspaper, radio, TV) and the web, including social media (e.g. Facebook, twitter) to encourage other institutions, patients and the public to discuss and adapt with lower barriers. Importantly, classical media (newspaper, radio, TV) are not to be neglected since (potential) patients and their relatives interested in our study are elderly and, thus, not familiar with novel web-media. The strong network of involved exponents from different professions will allow to broadly disseminate of our results from *module 1, 2* and *3* into the public.

2.1 Previous achievements in knowledge and technology transfer

Our previous research and clinical expertise comprises different elements important to the successful completion of the In-HospiTOOL-study. We have profound methodological know-how acquired through playing key roles in the conduct of various multicenter randomized controlled trials involving several Swiss and national institutions [46, 69, 86-89]. Results of these trials have had a profound impact on international guidelines. First, reduced corticosteroid use for patients with chronic obstructive pulmonary disease (COPD) exacerbation has been included in the recent GOLD guidelines [88]. Second, measurement of procalcitonin for

antibiotic decision making has been included in the recent surviving sepsis campaign guidelines [90, 91] and international respiratory medicine guidelines [46, 87, 92]. Third, corticosteroids have now been suggested for use in pneumonia patients based on a recent trial from our research network [69].

In addition to knowledge transfer from clinical trials, we have established a new insulin algorithm in our clinic work for inpatient management [93]. This algorithm is now integrated into our medical record system (KISIM, Cistec). It is also being used in the University Hospital Zürich and other Swiss hospitals using the same electronic medical record system.

2.2 Activities planned

Plans for dissemination

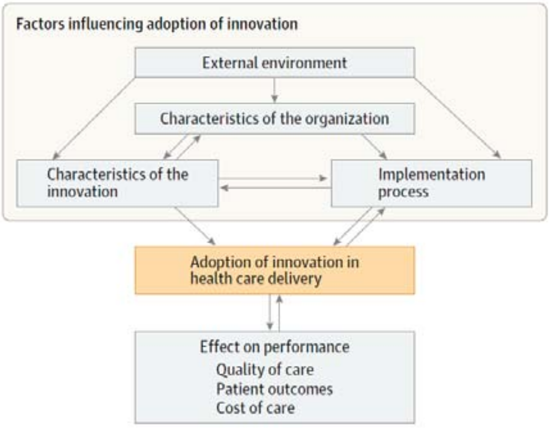

Figure 6. Framework for analyzing the adoption of health care innovations (adapted from [37]).

The use of data generated in this study, experience and networking of sounding board members (see section 2.3), and key elements of implementation science will improve health care, health systems, and finally patients` health. We will refer to tailored implementation interventions, being strategies that are designed to achieve desired changes in healthcare practice based on an assessment of determinants of healthcare practice [94]. Four main groups of variables that interact with adoption of innovations were previously identified (**Figure 6**) [95]: the external environment (e.g. new payment models), the structure of the organization (e.g. integrated delivery systems), the characteristics of the innovation (e.g., the strength of the evidence supporting it), and the processes used (e.g., bottom-up vs top-down decision making). This framework not only focuses on how to more quickly adopt and spread innovations that will benefit patients but also helps to understand how organizations eliminate treatments, practices, and policies that do not benefit patients (optimized resource allocation). Whether hospitals and health care institutions can do this better than others need to be identified. Health care delivery innovations such as the elements of the "In-HospiTOOL" focus on groups of patients defined by factors such as the site of care or the complexity of polymorbids` clinical situation. Although practicing physicians and the patients for whom they provide care are affected by these innovations, decision making about their use will be the primary responsibility of all involved practice managers and organizational leaders (stakeholders). This is crucial because of its complexity with diverse engaged individuals from different organizational levels and sometimes beyond. Existing evidence suggests that such innovations will have substantial potential to improve health care and reduce costs. Therefore, the implementation science framework can be used to identify the barriers to their successful implementation and strategies for overcoming them [96]. Furthermore, we must be aware of two important points: recognition that the increasing burden of chronic illness in the Swiss population cannot be addressed without engaging patients and their caregivers in effective self-care, behavior change, and chronic disease management; and the need to better align treatment choices with patients' well informed preferences and values through shared decision making. These changes in practice will implicate a fundamental change in the historical framework of the health care provider as expert and the patient as passive recipient.

As collaborators in the framework will be representatives from the hospitals (physicians, nurses, social workers, information scientists, controllers, etc.), general practitioner responsible for out-patient and pre-hospital setting (ARGOMED, largest Swiss managed care network of general practitioners), post-acute care facilities, health care authorities (DGS), health care insurances (santésuisse, curafutura), inter-professional board of health care management experts (Institute for Systemic Management and Public Governance, IMP-HSG (University St. Gallen)), and public benchmarking and internet providers (Nationaler Verein für Qualitätsentwicklung in Spitäler und Kliniken [ANQ], Spitalfinder, etc.). This will allow us built-up a – on purpose - heterogeneous sounding board involving all important stakeholders and authorities in a comprehensive health care process, including policies and financers. Based on this sounding board, we will be able to analyze external environment and organization characteristics, as we

know that complex interventions may work best if tailored to local circumstances rather than being completely standardized [97]. Doing so, adoption of the “In-HospiTOOL” will be facilitated by direct feedback of the stakeholders, enabling continuous immediately improvement of processes. Hence, long-term collaboration between hospitals and other health care providers will be strengthened, leading to an improved and step-less treatment continuum of chronic ill patients.
One major aim is to broadly disseminate our comprehensive patient-centered data, gathered in the observational as well as in the interventional phase. First, we intend to give patients and stakeholders a better understanding of the multifaceted health care processes in Switzerland. Knowledge about this elementary integrative health care process will translate into optimized transparency and education. In this context, we will publish data about resource use, patient outcome, patient satisfaction, functional status, and overall hospital costs; on the one hand in a public version at hospitals websites and on the other hand in diverse scientific journals as well as in patient brochures, issued by health care providers, health care insurances, and government. This will finally have a competitive influence on national-wide health care providers as well as on financiers and government that will open discussion about new recompense and payment strategies in this increasing polymorbid patient population. As already mentioned by the SNF, the “Wissens- und Technologie Transferstelle (WTT)” of the University Basel will play a crucial part in definition of dissemination strategies.

2.3 Implementation partners: references and contributions

Sounding Board of Participating Hospitals, Stakeholders and Government Authorities

In the past years we have built a multi-faceted network of pre-hospital, in-hospital and post-acute care partners. For this project, we will be advised by key academic, executive, administrative, clinical stakeholders and health care representatives.

I) General practitioner responsible for out-patient and pre-hospital setting
- ARGOMED, largest Swiss managed care network of general practitioners: CEO K. Züger, President Dr. W. Czerwenka

General practitioners will play an elementary part in improving transition of patients from the out-hospital to the in-hospital setting and *vice versa*. This will be an indispensable step in strategies of improving and simplifying data exchange.

II) Chief executives, physicians, nurses, social workers of participating hospitals
- University Hospital Basel: CEO Dr. W. Kübler, Prof. S. Bassetti, Prof. S. de Geest, Dr. J. Martin
- Kantonsspital Aarau: CEO Dr. R. Rhiner, Prof. B. Müller, Mrs. H. Weber
- Kantonsspital St. Gallen: CEO Dr. H. Germann, Prof. M. Brändle, Mrs. N. Mösli, Mrs. B. Schoop
- Luzerner Kantonsspital: CEO B. Fuchs, Prof. C. Henzen, Mr. M. Döring, Mr. D. Gralher
- Kantonsspital Fribourg (HFR): CEO Mrs. C. Käch, CMO Dr. I. Spicher

Leading authorities of their hospitals and disciplines provide full support in performing this study with a consecutively inclusion of medical in-patients and a large adherence in using the study templates (elements of the “In-HospiTOOL”).

III) Post-acute care facilities
- Klinik Barmelweid: CEO Mr. B. Stierlin, Dr. Thomas Sigrist
- Rehaklinik Bad Schinznach: CEO Mr. B. Schläfli, Dr. St. Bützberger
- Spitex of Canton of Aargau: Mr. H. R. Häny, Mrs. P. Baur
- Pflegeheim Lindenfeld: CEO Mr. T. Holliger, Dr. I. Amrhein, Mrs. D. Deubelbeiss

Post-acute care facilities will play an elementary part in improving transition of patients from the acute setting hospital to their institution. Based on the PACD, patients will be registered earlier in a post-acute care facility, enabling an optimal preparation at an earlier stage. Doing so, resources will be shared more efficiently. Personal contacts of institutions from the cantons Basel-Stadt, St. Gallen, Lucerne, and Fribourg pending upon funding of the proposal.

IV) Health care authorities

- Cantonal: Departement für Gesundheit und Soziales (DGS) AG: Mr. U. Zanoni, Head "MIVAG" (Masterplan Integrative Versorgung / eHealth Aargau)
- Federal: Bundesamt für Gesundheit (BAG), Bundesamt für Statistik (BFS)

We asked the BAG and BFS for access to nationwide patient- and hospital-centered data from registries. Government authorities will be significantly involved in wider implementation and dissemination of the studies` results. They will become important in defining new recompense and payment systems.

V) Health insurances
- selected health insurances (e.g. Helsana, CEO Mr. D. H. Schmutz, President Prof. T. Szucs is member of the Medical Faculty of the University of Basel as the PI of this grant)
- santésuisse, curafutura: (personal contacts pending upon funding of the proposal)

Health insurance authorities will be significantly involved in wider implementation and dissemination of the studies` results. They will become important in defining new recompense and payment systems together with government authorities.

VI) Public benchmarking and internet providers
- Nationaler Verein für Qualitätsentwicklung in Spitäler und Kliniken (ANQ): CEO Dr. P. Busch
- http://www.spitalfinder.ch/de/
- http://www.spitalinformation.ch

Public benchmarking and internet providers will have a supportive role in defining further patient-centered outcomes. They will give essential support in establishing a multi-health care institution data warehouse and in continuous benchmarking and optimizing strategies. Personal contacts pending upon funding of the proposal.

VII) Inter-professional Board of Health Care Management Experts
- Institute for Systemic Management and Public Governance, IMP-HSG (University St. Gallen), St. Gallen: Prof. J. Rüegg-Stürm (the PI is member of the "executive circle" led by Prof. Rüegg-Stürm)
- Department of Psychology, University of Berne: Fr. Prof. P. Perrig (ongoing thesis project)
- Department of Nursing Development and International Collaboration, Swiss Nursing Association: Mrs. R. Koch
- Universities of Applied Sciences, Department of Nursing; Winterthur, Prof. A. Koppitz; Berne, Prof. S. Hahn; St. Gallen, Prof. B. Senn

VIII) Statistical collaboration
- Harvard school of public health, Boston, USA: Prof. E. J. Orav (personal contact of Prof. Ph. Schütz, co-author of the grant application)

Using the expertise of several health care management experts we will define further secondary analyses of our study to generate a multi-facetted implementation strategy and a broad dissemination of our results.

2.4 Timetable and milestones

While performing the In-HospiTOOL-study we will broaden our sounding board that will facilitate wider implementation of our results. After obtaining first results, we will organize a sounding board meeting in the late 2019 to define further steps of dissemination, since namely in research - in our experience - learning is also a result of doing. Thereby, if feasible, additional ancillary analyses will be performed. Increasing the impact on the Swiss health care system. In this context, health care providers, health insurances and government authorities will publish patient-centered data about previously defined outcomes, starting in the first half of 2020. In addition, leading authorities of the hospitals will decide about a continuation of the "In-HospiTOOL". Benchmarking and continuous process optimizing will be performed analogously. After a further 1-year implementation and dissemination in previous or new hospitals, health insurances and government authorities will discuss new recompense and payment systems. Depending on Swiss long-term results international validation may be an approbate medium to finally introduce and fix the "In-HospiTOOL" in the (inter-)national health care system.

Objectives	Jan-Jun 2019	Jul-Dec 2019	Jan-Jun 2020	Jul-Dec 2020	Jan-Jun 2021	Jul-Dec 2021	Jan-Jun 2022	Jul-Dec 2022
**Preparation of wider dissemination and implementation**								
Sounding board meeting, preparation		■	■	■				
Patient-centered data publishing			■	■	■	■	■	■
**Secondary analyses as preparation for national-wide dissemination**								
Data analyses	■	■	■	■	■	■	■	■
Manuscript preparation and publication			■	■	■	■	■	■
**Administration**								
Benchmarking, monitoring, process optimizing	■	■	■	■	■	■	■	■
**Dissemination, national-wide implementation**								
Stakeholder meeting, consensus conference, preparation*				■	■	■	■	■
**Requested funding period (4 years)**	■	■	■	■				
**Additional funding request for further dissemination**				■	■			

Table 2. Schedule and milestones of the implementation and dissemination strategy of “In-HospiTOOL” findings.

3. Significance

3.1 Scientific significance

Clinical trials that are embedded into usual care (“comprehensive effectiveness research”) have the potential to yield outcomes of great relevance to the institutions where they are performed and at the same time to yield information that may be generalizable to the health care system at large [85].

Health care costs in Switzerland are high and rising due to the aging, polymorbid population. Scientific evidence regarding performance, safety and cost-effectiveness of specific integrative multi-professional care models tailored to the Swiss health care system is largely lacking. The “In-HospiTOOL” is an integrative multi-professional inpatient management tool that enables a better understanding of the multifaceted health care processes and will close this gap. Through a standardized but at the same time individualized approach, it will improve the inter-professional management of patients from ED admission to hospital discharge to home or a nursing care facility. This will translate into optimized transparency, resource use, patient outcome and satisfaction, functional status, and overall hospital costs. We expect that the results of the In-HospiTOOL-study will be widely, directly and rapidly applied – and indeed, will contribute to a new standard of (inter-)national health care.

In addition to the main interventional trial, gathering of data from around ~45`000 patients from at least 5 Swiss hospitals will help to establishing a national-wide framework involving important stakeholder of the Swiss health care system. Networking is a prerequisite for improving sustainable patient-centered health care delivery with an optimal resource allocation. This will lead to a more efficient patient flow with decreased risk for hospital associated adverse outcomes. Also, the large dataset will allow to compare different outcomes of different patient populations across different hospitals with each individual health care strategies. We will also be open to share our data with other national health care researchers for secondary analyses. In addition, health insurance and policy authorities will largely profit from these data to conceptualize new reimbursement strategies in the polymorbid inpatient setting.

Such embedded comparative effectiveness research relies on the engagement of care providers and health care systems as active partners in defining the objectives of the research rather than as passive consumers of its product [85]. This pragmatic research will enforce rethinking and redefining traditional ethical and regulatory standards (including informed consent and engagement in research) in this paradigm of low risk.

3.2 Social and economic significance

Many patients are cared for by their relatives and families, putting a large strain on them. In Switzerland, this unpaid care of adult patients was accounted for 2`414 Mio. Swiss francs in

2013 [98]. Comprehensive discharge planning without family support is unlikely to be possible, thus, early involvement of relatives is inevitable. Herein, the “In-HospiTOOL” will play an important role, by enabling an early inter-professional communication including patient and relatives, which is fundamental in an optimized discharge planning. In addition, by creating transparency of the entire health care process, polymorbid patients and their relatives will be better informed about relevant, patient-centered outcome measures including satisfaction, enhancing hospitals` contest in improving health care quality [77].
Our study will have important implication in generating evidence in this new research field. In terms of a “smarter medicine” the “In-HospiTOOL” rigorously uncovers health care service misuse in this large and complex polymorbid patient population and will serve as a milestone in establishment of an improved patient flow. Our project will animate new generation health care personnel and researchers to actively participate in defining novel strategies to sustainably increase patients` safety without further cost explosion.
Given the continuous aging of the Swiss population [99], the proportion of polymorbid patients will further rise, and traditional health care models are no longer suitable for this challenge as they are still designed for mono-morbid patients [100]. In consequence, expenses are reaching new levels yearly, with a national increase from 10.3% of gross domestic product (GDP) (62.5 billion Swiss francs) to 11.1% of GDP (71.2 billion Swiss francs) within 2010 and 2014 [101]. Daily costs in an average Swiss somatic hospital were 1’690 Swiss francs in 2011. In 2011, the average hospital stay was 7.5 days with 1.36 million hospitalizations [102]. Herein, the “In-HospiTOOL” will support containment of health care expanses in the in-hospital setting, mainly due to identification and reduction of avoidable resource misuse. As outlined above, interventions in the Medical University Clinic of Kantonsspital Aarau have already reduced length of stay by approximately 3 days as compared to other tertiary care hospitals in Switzerland [69]. Envisioning that similar improvements will be achieved by implementation of “In-HospiTOOL” in other hospitals, we assume that costs may drop also on a nation-wide level.

4. References mentioned in this proposal (own research in **bold**)

1. Barnett, K., et al., *Epidemiology of multimorbidity and implications for health care, research, and medical education: a cross-sectional study.* Lancet, 2012. **380**(9836): p. 37-43.
2. Mercer, S.W., et al., *Multimorbidity and the inverse care law in primary care.* BMJ, 2012. **344**: p. e4152.
3. Bahler, C., et al., *Multimorbidity, health care utilization and costs in an elderly community-dwelling population: a claims data based observational study.* BMC Health Serv Res, 2015. **15**: p. 23.
4. Kadam, U.T., et al., *Chronic disease multimorbidity transitions across healthcare interfaces and associated costs: a clinical-linkage database study.* BMJ Open, 2013. **3**(7).
5. Selby, K. and S. Edwards, *Time-Based Billing: What Primary Care in the United States Can Learn From Switzerland.* JAMA Intern Med, 2016.
6. Biller-Andorno, N. and T. Zeltner, *Individual Responsibility and Community Solidarity--The Swiss Health Care System.* N Engl J Med, 2015. **373**(23): p. 2193-7.
7. Boyd, C.M., et al., *Clinical practice guidelines and quality of care for older patients with multiple comorbid diseases: implications for pay for performance.* JAMA, 2005. **294**(6): p. 716-24.
8. Hughes, L.D., M.E. McMurdo, and B. Guthrie, *Guidelines for people not for diseases: the challenges of applying UK clinical guidelines to people with multimorbidity.* Age Ageing, 2013. **42**(1): p. 62-9.
9. Roland, M. and C. Paddison, *Better management of patients with multimorbidity.* BMJ, 2013. **346**: p. f2510.
10. O'Leary, K.J., et al., *Interdisciplinary teamwork in hospitals: a review and practical recommendations for improvement.* J Hosp Med, 2012. **7**(1): p. 48-54.
11. Huang, C., et al., *Implementation of proven interventions in general medical inpatients: development and evaluation of a new quality indicator for drug therapy.* Qual Saf Health Care, 2008. **17**(4): p. 269-74.
12. Classen, D.C., et al., *'Global trigger tool' shows that adverse events in hospitals may be ten times greater than previously measured.* Health Aff (Millwood), 2011. **30**(4): p. 581-9.
13. Sabbatini, A.K., et al., *In-Hospital Outcomes and Costs Among Patients Hospitalized During a Return Visit to the Emergency Department.* JAMA, 2016. **315**(7): p. 663-71.
14. Hogan, H., et al., *Preventable deaths due to problems in care in English acute hospitals: a retrospective case record review study.* BMJ Qual Saf, 2012. **21**(9): p. 737-45.
15. Hsia, R.Y., A.B. Friedman, and M. Niedzwiecki, *Urgent Care Needs Among Nonurgent Visits to the Emergency Department.* JAMA Intern Med, 2016.
16. Le, S.T. and R.Y. Hsia, *Timeliness of care in US emergency departments: an analysis of newly released metrics from the Centers for Medicare & Medicaid services.* JAMA Intern Med, 2014. **174**(11): p. 1847-9.

17. Lindsay, P., et al., *The development of indicators to measure the quality of clinical care in emergency departments following a modified-delphi approach.* Acad Emerg Med, 2002. **9**(11): p. 1131-9.
18. Schull, M.J., et al., *Prioritizing performance measurement for emergency department care: consensus on evidence-based quality of care indicators.* Cjem, 2011. **13**(5): p. 300-9, E28-43.
19. Kulstad, E.B., et al., *ED overcrowding is associated with an increased frequency of medication errors.* Am J Emerg Med, 2010. **28**(3): p. 304-9.
20. Liu, S.W., et al., *A pilot study examining undesirable events among emergency department-boarded patients awaiting inpatient beds.* Ann Emerg Med, 2009. **54**(3): p. 381-5.
21. Louis Simonet, M., et al., *A predictive score to identify hospitalized patients' risk of discharge to a post-acute care facility.* BMC Health Serv Res, 2008. **8**: p. 154.
22. Fox, M.T., et al., *Effectiveness of early discharge planning in acutely ill or injured hospitalized older adults: a systematic review and meta-analysis.* BMC Geriatr, 2013. **13**: p. 70.
23. McMartin, K., *Discharge planning in chronic conditions: an evidence-based analysis.* Ont Health Technol Assess Ser, 2013. **13**(4): p. 1-72.
24. Fox, M., *Nurse-led early discharge planning for chronic disease reduces hospital readmission rates and all-cause mortality.* Evid Based Nurs, 2016. **19**(2): p. 62.
25. Kripalani, S., et al., *Effect of a pharmacist intervention on clinically important medication errors after hospital discharge: a randomized trial.* Ann Intern Med, 2012. **157**(1): p. 1-10.
26. Kripalani, S., et al., *Deficits in communication and information transfer between hospital-based and primary care physicians: implications for patient safety and continuity of care.* JAMA, 2007. **297**(8): p. 831-41.
27. Coleman, E.A., et al., *Understanding and execution of discharge instructions.* Am J Med Qual, 2013. **28**(5): p. 383-91.
28. Verhaegh, K.J., et al., *Transitional care interventions prevent hospital readmissions for adults with chronic illnesses.* Health Aff (Millwood), 2014. **33**(9): p. 1531-9.
29. Coleman, E.A., et al., *The care transitions intervention: results of a randomized controlled trial.* Arch Intern Med, 2006. **166**(17): p. 1822-8.
30. Naylor, M.D., et al., *Transitional care of older adults hospitalized with heart failure: a randomized, controlled trial.* J Am Geriatr Soc, 2004. **52**(5): p. 675-84.
31. Buurman, B.M., et al., *Comprehensive Geriatric Assessment and Transitional Care in Acutely Hospitalized Patients: The Transitional Care Bridge Randomized Clinical Trial.* JAMA Intern Med, 2016. **176**(3): p. 302-9.
32. Jack, B.W., et al., *A reengineered hospital discharge program to decrease rehospitalization: a randomized trial.* Ann Intern Med, 2009. **150**(3): p. 178-87.
33. Pannick, S., et al., *Effects of Interdisciplinary Team Care Interventions on General Medical Wards: A Systematic Review.* JAMA Intern Med, 2015. **175**(8): p. 1288-98.
34. Ellis, G., et al., *Comprehensive geriatric assessment for older adults admitted to hospital: meta-analysis of randomised controlled trials.* BMJ, 2011. **343**: p. d6553.
35. Washington, D.I.o.M., *Measuring the Impact on Interprofessional Education on Collaborative Practice and Patient Outcomes.* 2015.
36. Chambers, D.A., W.G. Feero, and M.J. Khoury, *Convergence of Implementation Science, Precision Medicine, and the Learning Health Care System: A New Model for Biomedical Research.* JAMA, 2016. **315**(18): p. 1941-2.
37. Fisher, E.S., S.M. Shortell, and L.A. Savitz, *Implementation Science: A Potential Catalyst for Delivery System Reform.* JAMA, 2016. **315**(4): p. 339-40.
38. Cassel, C.K., et al., *Getting more performance from performance measurement.* N Engl J Med, 2014. **371**(23): p. 2145-7.
39. Higgins, A., G. Veselovskiy, and L. McKown, *Provider performance measures in private and public programs: achieving meaningful alignment with flexibility to innovate.* Health Aff (Millwood), 2013. **32**(8): p. 1453-61.
40. Meyer, G.S., et al., *More quality measures versus measuring what matters: a call for balance and parsimony.* BMJ Qual Saf, 2012. **21**(11): p. 964-8.
41. Jha, A.K., E.J. Orav, and A.M. Epstein, *Public reporting of discharge planning and rates of readmissions.* N Engl J Med, 2009. **361**(27): p. 2637-45.
42. Moore, L., et al., *Derivation and Validation of a Quality Indicator to Benchmark In-Hospital Complications Among Injury Admissions.* JAMA Surg, 2016.
43. Taichman, D.B., et al., *Sharing Clinical Trial Data--A Proposal from the International Committee of Medical Journal Editors.* N Engl J Med, 2016. **374**(4): p. 384-6.
44. Bierer, B.E., et al., *A Global, Neutral Platform for Sharing Trial Data.* N Engl J Med, 2016.
45. Merson, L., O. Gaye, and P.J. Guerin, *Avoiding Data Dumpsters - Toward Equitable and Useful Data Sharing.* N Engl J Med, 2016.
46. **Schuetz, P., et al., *Effect of procalcitonin-based guidelines vs standard guidelines on antibiotic use in lower respiratory tract infections: the ProHOSP randomized controlled trial.* JAMA, 2009. 302(10): p. 1059-66.**
47. **Schuetz, P., et al., *Quality of care delivered by fee-for-service and DRG hospitals in Switzerland in patients with community-acquired pneumonia.* Swiss Med Wkly, 2011. 141: p. w13228.**

48. **Suter-Widmer, I., et al., *Predictors for length of hospital stay in patients with community-acquired pneumonia: results from a Swiss multicenter study.* BMC Pulm Med, 2012. 12: p. 21.**
49. **Baehni, C., et al., *Which patients with lower respiratory tract infections need inpatient treatment? Perceptions of physicians, nurses, patients and relatives.* BMC Pulm Med, 2010. 10: p. 12.**
50. **Faessler, L., et al., *Psychological distress in medical patients seeking ED care for somatic reasons: results of a systematic literature review.* Emerg Med J, 2015.**
51. **Albrich, W.C., et al., *Biomarker-enhanced triage in respiratory infections: a proof-of-concept feasibility trial.* Eur Respir J, 2013. 42(4): p. 1064-75.**
52. **Albrich, W.C., et al., *Enhancement of CURB65 score with proadrenomedullin (CURB65-A) for outcome prediction in lower respiratory tract infections: derivation of a clinical algorithm.* BMC Infect Dis, 2011. 11: p. 112.**
53. **Conca, A., et al., *OPTIMA – Optimierter Patienten-Transfer durch innovatives multidisziplinäres Assessment - Projektbeschreibung der Phase I.* Zeitschrift für Pflegewissenschaft 2012:291-298, 2012: p. 291-298.**
54. **Katan, M., et al., *Stress hormones predict cerebrovascular re-events after transient ischemic attacks.* Neurology, 2011. 76(6): p. 563-6.**
55. **Dusemund, F., et al., *Multidisciplinary Assessment to Personalize Length of Stay in Acute Decompensated Heart Failure (OPTIMA II ADHF).* J Clin Med Res, 2012. 4(6): p. 402-9.**
56. **Birsen Arici, M.M., Philipp Schuetz, Beat Mueller, Werner C. Albrich, *Potential Role of Biomarkers in the Management of Syncope.* International Journal of Clinical Medicine, 2012(3): p. 744-750.**
57. **Drozdov, D., et al., *Procalcitonin, pyuria and proadrenomedullin in the management of urinary tract infections--'triple p in uti': study protocol for a randomized controlled trial.* Trials, 2013. 14: p. 84.**
58. **Drozdov, D., et al., *Procalcitonin and pyuria-based algorithm reduces antibiotic use in urinary tract infections: a randomized controlled trial.* BMC Med, 2015. 13: p. 104.**
59. Karakoumis, J., et al., *Emergency Presentations With Nonspecific Complaints-the Burden of Morbidity and the Spectrum of Underlying Disease: Nonspecific Complaints and Underlying Disease.* Medicine (Baltimore), 2015. **94**(26): p. e840.
60. **Conca, A., et al., *[At admission planning discharge already].* Krankenpflege. Soins infirmiers, 2013. 106(1): p. 20-3.**
61. **Conca, A., et al., *Erfassung eines Nachakutpflegebedarf bei hospitalisierten, medizinischen Patienten durch die „Post-Acute Care Discharge scores" (PACD).* Pflegewissenschaft, 2015. 11/15, 17. Jahrgang.**
62. Griffiths, P. and J. Wilson-Barnett, *The effectiveness of 'nursing beds': a review of the literature.* J Adv Nurs, 1998. **27**(6): p. 1184-92.
63. Griffiths, P.D., et al., *Effectiveness of intermediate care in nursing-led in-patient units.* Cochrane Database Syst Rev, 2007(2): p. CD002214.
64. **Brunner, C., et al., *Patienten und pflegende Angehörige im Fokus von Nurse-led care.* Krankenpflege, 2015. 1: p. 14-17.**
65. Griffiths, P., et al., *Post-acute intermediate care in nursing-led units: a systematic review of effectiveness.* Int J Nurs Stud, 2005. **42**(1): p. 107-16.
66. Harris, R., et al., *Economic evaluation of a nursing-led inpatient unit: the impact of findings on management decisions of service utility and sustainability.* J Nurs Manag, 2005. **13**(5): p. 428-38.
67. **Schuetz, P., et al., *Optimizing triage and hospitalization in adult general medical emergency patients: the triage project.* BMC Emerg Med, 2013. 13: p. 12.**
68. Paasche-Orlow, M.K., et al., *Tailored education may reduce health literacy disparities in asthma self-management.* Am J Respir Crit Care Med, 2005. **172**(8): p. 980-6.
69. **Blum, C.A., et al., *Adjunct prednisone therapy for patients with community-acquired pneumonia: a multicentre, double-blind, randomised, placebo-controlled trial.* Lancet, 2015. 385(9977): p. 1511-8.**
70. **Schuetz, P., et al., *Biomarkers from distinct biological pathways improve early risk stratification in medical emergency patients: the multinational, prospective, observational TRIAGE study.* Crit Care, 2015. 19(1): p. 377.**
71. **Christ, M., et al., *Modern triage in the emergency department.* Deutsches Arzteblatt international, 2010. 107(50): p. 892-8.**
72. **Steiner, D., et al., *Performance of the Manchester Triage System in Adult Medical Emergency Patients: A Prospective Cohort Study.* J Emerg Med, 2015.**
73. **Kutz, A., et al., *Predictors for Delayed Emergency Department Care in Medical Patients with Acute Infections - An International Prospective Observational Study.* PLoS One, 2016. 11(5): p. e0155363.**
74. **Felder, S., et al., *Association of nutritional risk and adverse medical outcomes across different medical inpatient populations.* Nutrition, 2015. 31(11-12): p. 1385-93.**
75. **Rast, A.C., et al., *Procalcitonin improves the Glasgow Prognostic Score for outcome prediction in emergency patients with cancer: a cohort study.* Dis Markers, 2015. 2015: p. 795801.**

76. Graedel, L., et al., *Vitamin D Deficiency Strongly Predicts Adverse Medical Outcome Across Different Medical Inpatient Populations: Results From a Prospective Study.* Medicine (Baltimore), 2016. 95(19): p. e3533.

77. Panzer, R.J., et al., *Increasing demands for quality measurement.* JAMA, 2013. **310**(18): p. 1971-80.

78. Jumbe, N.L., J.C. Murray, and S. Kern, *Data Sharing and Inductive Learning - Toward Healthy Birth, Growth, and Development.* N Engl J Med, 2016.

79. Baker, R., et al., *Tailored interventions to overcome identified barriers to change: effects on professional practice and health care outcomes.* Cochrane Database Syst Rev, 2010(3): p. CD005470.

80. *14 - Internationale Klassifikation der Krankheiten (ICD-10). http://www.bfs.admin.ch/bfs/portal/de/index/infothek/nomenklaturen/blank/blank/cim10/02/05.html. Accessed 27.05.2016.* Bundesamt für Statistik: Neuchâtel, 2014.

81. Elixhauser, A., et al., *Comorbidity measures for use with administrative data.* Med Care, 1998. **36**(1): p. 8-27.

82. Zuckerman, R.B., et al., *Readmissions, Observation, and the Hospital Readmissions Reduction Program.* N Engl J Med, 2016. **374**(16): p. 1543-51.

83. Penfold, R.B. and F. Zhang, *Use of interrupted time series analysis in evaluating health care quality improvements.* Acad Pediatr, 2013. **13**(6 Suppl): p. S38-44.

84. Powell, B.J., et al., *A compilation of strategies for implementing clinical innovations in health and mental health.* Med Care Res Rev, 2012. **69**(2): p. 123-57.

85. Fiore, L.D. and P.W. Lavori, *Integrating Randomized Comparative Effectiveness Research with Patient Care.* N Engl J Med, 2016. **374**(22): p. 2152-8.

86. Albrich, W.C., et al., *Biomarker-enhanced triage in respiratory infections - a proof-of-concept feasibility trial.* Eur Respir J, 2013.

87. Briel, M., et al., *Procalcitonin-guided antibiotic use vs a standard approach for acute respiratory tract infections in primary care.* Arch Intern Med, 2008. 168(18): p. 2000-7; discussion 2007-8.

88. Leuppi, J.D., et al., *Short-term vs conventional glucocorticoid therapy in acute exacerbations of chronic obstructive pulmonary disease: the REDUCE randomized clinical trial.* JAMA, 2013. 309(21): p. 2223-31.

89. Thomann, R., et al., *Evaluation of an algorithm for intensive subcutaneous insulin therapy in noncritically ill hospitalised patients with hyperglycaemia in a randomised controlled trial.* Swiss Med Wkly, 2013. 143: p. w13808.

90. Schuetz, P., et al., *Procalcitonin to guide initiation and duration of antibiotic treatment in acute respiratory infections: an individual patient data meta-analysis.* Clin Infect Dis, 2012. 55(5): p. 651-62.

91. Dellinger, R.P., et al., *Surviving sepsis campaign: international guidelines for management of severe sepsis and septic shock: 2012.* Crit Care Med, 2013. **41**(2): p. 580-637.

92. Woodhead, M., et al., *Guidelines for the management of adult lower respiratory tract infections.* Clin Microbiol Infect, 2011. **17(Suppl. 6): E1–E59**.

93. Schuetz P, B.N., German S, Baumgartner A, Mueller B, *Incorporation of Nutritional Factors and Insulin Resistance into Insulin Sliding Scales in the Non-Critical Care Inpatient Setting: A "Before/After" Study.* J Diabetes Metab, 2014. 5(1).

94. Wensing, M., M. Bosch, and R. Grol, *Developing and selecting interventions for translating knowledge to action.* Cmaj, 2010. **182**(2): p. E85-8.

95. Damschroder, L.J., et al., *Fostering implementation of health services research findings into practice: a consolidated framework for advancing implementation science.* Implement Sci, 2009. **4**: p. 50.

96. Milstein, A. and S. Shortell, *Innovations in care delivery to slow growth of US health spending.* JAMA, 2012. **308**(14): p. 1439-40.

97. Craig, P., et al., *Developing and evaluating complex interventions: the new Medical Research Council guidance.* BMJ, 2008. **337**: p. a1655.

98. *Schweizerische Arbeitskräfteerhebung (SAKE): Modul Unbezahlte Arbeit und Schweizerische Lohnstrukturerhebung (LSE). Accessed 27.06.2016.*, 2013, Bundesamt für Statistik: Neuchâtel.

99. *Statistisches Lexion der Schweiz: Durchschnittliche Lebensdauer in Jahren. Accessed 29.06.2016.*, 2016, Bundesamt für Statistik: Neuchâtel.

100. Céline Schmid Botkine, K.B., Ewa Kasprzycki, Fabienne Rausa, *Demos Newsletter: Korrigierte Version vom 21.7.2010.* Bundesamt für Statistik, 2010(Nr. 1): p. 1–15.

101. *Kosten und Finanzierung des Gesundheitswesens 2014: Provisorische Zahlen: Medienmitteilung. Accessed 28.06.2016.*, 2016, Bundesamt für Statistik: Neuchâtel.

102. *Statistiken der stationären Gesundheitsversorgung 2011: Provisorische Zahlen: 2011 behandelten Schweizer Spitäler 36'853 Personen pro Tag. Accessed 27.06.2016.*, 2012, Bundesamt für Statistik: Neuchâtel.
