# Supplementary material for: Association of Interprofessional Discharge Planning Using an Electronic Health Record Tool With Hospital Length of Stay Among Patients with Multimorbidity: A Nonrandomized Controlled Trial
Source: JAMA Netw Open. 2022 Sep 28;5(9):e2233667. doi: 10.1001/jamanetworkopen.2022.33667 (PMC9520366; doi:10.1001/jamanetworkopen.2022.33667)
Supplement: Supplement 2. — eTable. Crude Estimates of Length of Stay, 30-Day Hospital Readmission, In-Hospital Mortality, and Facility Discharge Among Intervention and Control Hospitals eFigure 1. Timeline and Study Design eFigure 2. Study Flow Diagram eFigure 3. Frequency of Tool Use Among Physicians and Nurses [file jamanetwopen-e2233667-s002.pdf]

Supplemental Online Content

Kutz A, Koch D, Haubitz S, et al. Association of interprofessional discharge planning using an electronic health record tool with hospital length of stay among patients with multimorbidity: a nonrandomized controlled trial. *JAMA Netw Open.* 2022;5(9):e2233667. doi:10.1001/jamanetworkopen.2022.33667

eTable. Crude Estimates of Length of Stay, 30-Day Hospital Readmission, In-Hospital Mortality, and Facility Discharge Among Intervention and Control Hospitals

eFigure 1. Timeline and Study Design

eFigure 2. Study Flow Diagram

eFigure 3. Frequency of Tool Use Among Physicians and Nurses

This supplemental material has been provided by the authors to give readers additional information about their work.

© 2022 Kutz A et al. *JAMA Network Open.*

eTable. Crude Estimates of Length of Stay, 30-Day Hospital Readmission, In-Hospital Mortality, and Facility Discharge Among Intervention and Control Hospitals

**Outcomes**	**Study group**	**Pre-intervention phase**												**Intervention phase**											
		**Feb. 17**	**Mar. 17**	**Apr. 17**	**May 17**	**Jun. 17**	**Jul. 17**	**Aug. 17**	**Sep. 17**	**Oct. 17**	**Nov. 17**	**Dec. 17**	**Jan. 18**	**Feb. 18**	**Mar. 18**	**Apr. 18**	**May 18**	**Jun. 18**	**Jul. 18**	**Aug. 18**	**Sep. 18**	**Oct. 18**	**Nov. 18**	**Dec. 18**	**Jan. 19**
**Hospital length of stay, mean (SD), days**	**Control**	7.7 (7.3)	7.6 (7.6)	7.6 (7.5)	7.6 (7.5)	7.5 (7.5)	7.4 (7.6)	7.5 (7.4)	7.4 (7.3)	7.4 (7.3)	7.5 (7.3)	7.6 (7.7)	7.5 (7.3)	7.5 (7.2)	7.5 (7.1)	7.4 (7.4)	7.4 (7.4)	7.2 (7.1)	7.3 (7.3)	7.3 (7.3)	7.2 (7.2)	7.5 (7.3)	7.4 (7.0)	7.4 (7.3)	7.6 (7.4)
	**Intervention**	7.7 (7.3)	7.5 (7.4)	7.6 (7.0)	7.6 (7.6)	7.8 (7.6)	7.6 (7.4)	7.7 (6.8)	7.9 (7.5)	7.8 (7.1)	7.5 (6.8)	7.2 (6.3)	7.4 (6.4)	7.5 (7.2)	7.7 (7.3)	7.5 (7.0)	7.5 (7.6)	7.2 (7.1)	7.6 (7.6)	7.1 (6.9)	7.3 (7.1)	7.3 (6.7)	7.3 (6.6)	6.9 (5.8)	6.8 (6.1)
**30-day hospital readmission, % (SD)**	**Control**	9.8 (11.7)	10.3 (12.3)	10.0 (12.6)	10.5 (12.6)	10.5 (13.0)	10.1 (12.5)	10.2 (12.4)	10.2 (12.7)	10.4 (12.7)	10.6 (12.9)	11.4 (12.2)	10.0 (11.8)	10.3 (12.5)	10.0 (12.0)	10.0 (12.2)	10.6 (12.3)	10.6 (13.2)	10.5 (12.6)	10.6 (12.7)	10.5 (12.9)	10.5 (12.3)	10.5 (12.7)	11.0 (12.6)	9.8 (12.0)
	**Intervention**	9.9 (11.2)	10.3 (11.3)	9.1 (12.5)	10.3 (12.3)	10.7 (12.5)	11.3 (13.7)	10.8 (13.3)	10.4 (13.3)	9.7 (11.9)	9.5 (13.2)	10.7 (13.2)	10.2 (11.0)	9.7 (11.3)	9.1 (10.8)	10.5 (13.4)	9.3 (11.6)	10.7 (13.5)	10.5 (13.2)	10.0 (11.5)	10.4 (13.0)	11.2 (12.9)	9.6 (11.6)	10.6 (12.3)	9.4 (12.1)
**In-hospital mortality, % (SD)**	**Control**	5.7 (10.9)	5.0 (10.3)	5.3 (10.2)	5.2 (10.0)	4.8 (10.1)	4.8 (9.9)	4.9 (10.6)	5.1 (10.3)	4.9 (10.1)	5.6 (10.6)	5.2 (9.9)	5.4 (10.4)	5.2 (10.2)	5.1 (10.1)	5.1 (10.0)	5.3 (10.2)	4.6 (9.8)	5.0 (10.1)	4.6 (9.7)	5.0 (10.1)	5.0 (9.8)	5.4 (10.4)	5.3 (10.3)	5.4 (10.1)
	**Intervention**	4.9 (9.4)	4.6 (10.0)	4.9 (11.6)	4.6 (10.3)	4.9 (10.6)	4.0 (8.9)	4.3 (9.9)	5.0 (10.7)	4.4 (10.0)	5.0 (10.9)	5.9 (12.1)	4.6 (10.0)	5.1 (9.3)	4.2 (9.5)	4.9 (11.6)	4.9 (10.0)	5.0 (10.2)	4.3 (8.7)	4.9 (10.2)	4.1 (9.8)	5.5 (11.0)	4.6 (10.2)	5.1 (10.7)	6.0 (12.1)
**Facility discharge, % (SD)**	**Control**	18.7 (23.1)	18.5 (23.1)	17.5 (22.9)	18.5 (23.5)	18.0 (22.9)	18.6 (23.7)	18.3 (23.5)	18.2 (23.0)	18.2 (23.1)	17.8 (23.0)	18.1 (22.4)	19.6 (22.7)	19.4 (23.6)	19.3 (23.1)	18.4 (23.1)	18.4 (23.4)	18.3 (23.4)	18.6 (23.2)	18.9 (23.8)	18.4 (23.0)	18.9 (23.3)	18.4 (23.2)	18.5 (22.9)	19.0 (22.8)
	**Intervention**	21.5 (26.4)	20.4 (25.0)	20.0 (6.2)	19.1 (25.0)	19.1 (24.5)	19.1 (25.5)	21.7 (25.8)	21.3 (26.1)	22.6 (25.8)	19.4 (25.7)	17.6 (24.7)	21.5 (25.7)	20.5 (25.7)	21.6 (24.7)	21.5 (27.2)	19.6 (25.1)	19.8 (26.2)	20.7 (25.6)	19.6 (25.1)	20.5 (26.6)	20.5 (26.1)	19.8 (24.5)	18.8 (25.5)	21.0 (26.0)

SD, standard deviation.

© 2022 Kutz A et al. *JAMA Network Open.*

eFigure 1. Timeline and Study Design

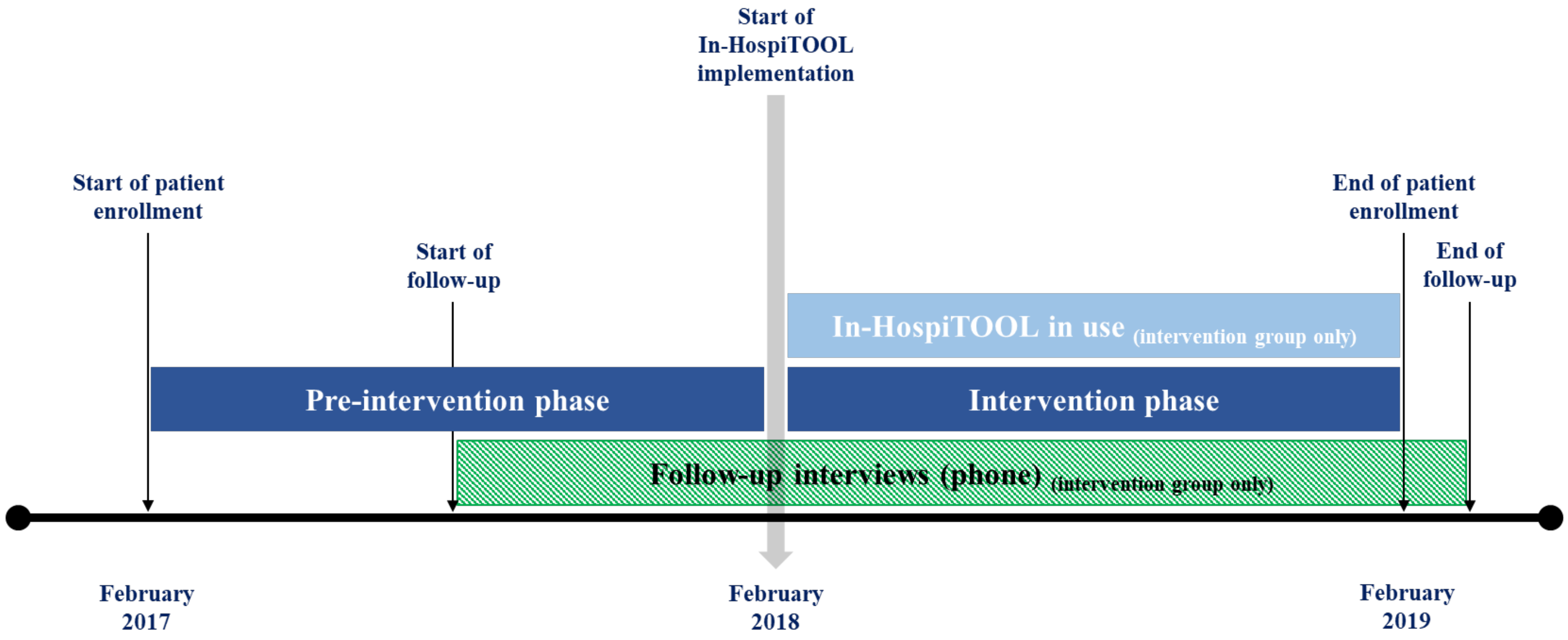

Illustration of the interrupted time series design used in this study. The diagram shows the implementation of an electronic interprofessional-led discharge planning tool (In-HospiTOOL) versus usual care in February 2018 to assess the effect and safety on hospital length of stay, in-hospital mortality, 30-day hospital readmission, and facility discharge. Follow-up phone interviews were performed among intervention hospitals only between August 2017 through end of February 2019.

© 2022 Kutz A et al. *JAMA Network Open.*

eFigure 2. Study Flow Diagram

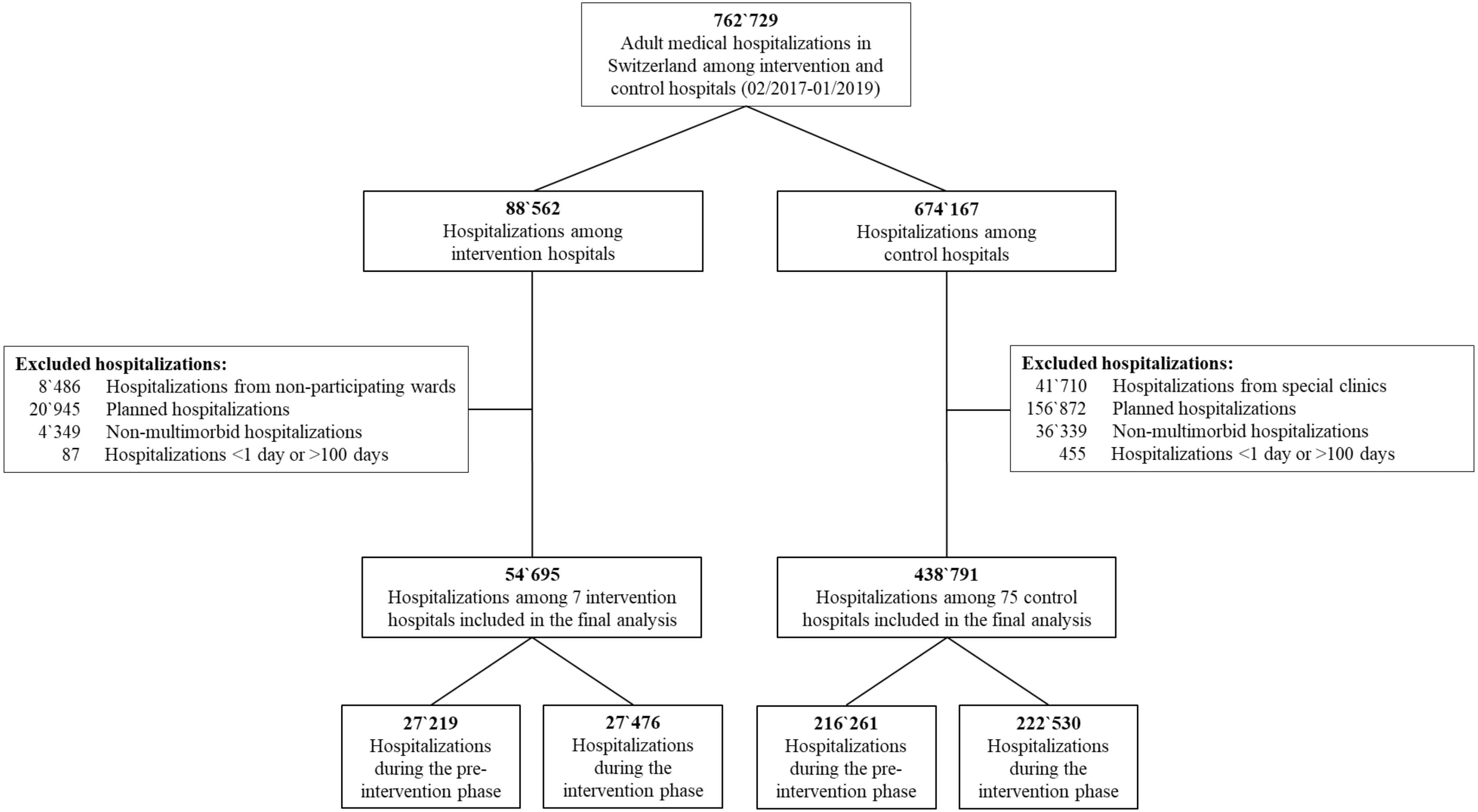

© 2022 Kutz A et al. *JAMA Network Open.*

eFigure 3. Frequency of Tool Use Among Physicians and Nurses

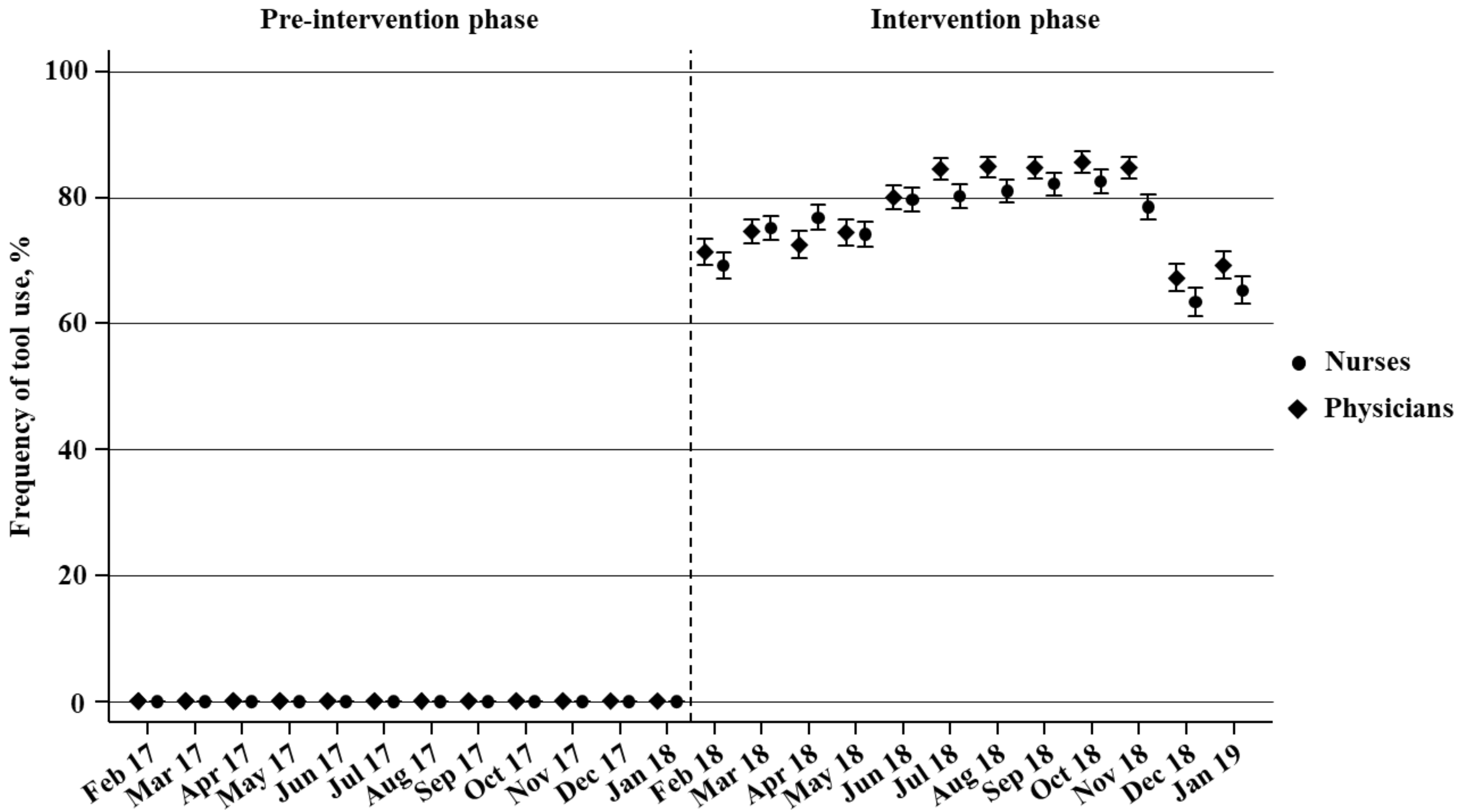

Frequency of tool use for physicians and nurses among intervention hospitals during the pre-intervention and intervention phase.

© 2022 Kutz A et al. *JAMA Network Open.*

© 2022 Kutz A et al. *JAMA Network Open.*
