# Supplementary material for: Association of Interprofessional Discharge Planning Using an Electronic Health Record Tool With Hospital Length of Stay Among Patients with Multimorbidity: A Nonrandomized Controlled Trial
Source: JAMA Netw Open. 2022 Sep 28;5(9):e2233667. doi: 10.1001/jamanetworkopen.2022.33667 (PMC9520366; doi:10.1001/jamanetworkopen.2022.33667)
Supplement: Supplement 3. — Data Sharing Statement [file jamanetwopen-e2233667-s003.pdf]

Data Sharing Statement

Kutz. Association of Interprofessional Discharge Planning Using an Electronic Health Record Tool With Hospital Length of Stay Among Patients with Multimorbidity. *JAMA Netw Open*. Published September 28, 2022. doi:10.1001/jamanetworkopen.2022.33667

Data
Data available: No
